# *Schistosoma mansoni* Adult Worm Protective and Diagnostic Proteins in *n*-Butanol Extracts Revealed by Proteomic Analysis

**DOI:** 10.3390/pathogens11010022

**Published:** 2021-12-24

**Authors:** Guidenn Sulbarán, Giovani C. Verissimo da Costa, Sandra Losada, José M. Peralta, Italo M. Cesari

**Affiliations:** 1 Laboratorio de Inmunoparasitología, Centro de Microbiología y Biología Celular, Instituto Venezolano de Investigaciones Científicas (IVIC), Caracas 1020, Venezuela; gsulbaranmachado@ibs.fr; 2 Departamento de Ciências Básicas, Instituto de Saúde de Nova Friburgo, Universidade Federal Fluminense (UFF), Nova Friburgo 28625-650, Brazil; giovaniverissimo@id.uff.br; 3 Sección de Biohemintiasis, Instituto de Medicina Tropical, Universidad Central de Venezuela (UCV), Caracas 1020, Venezuela; sandra.losada@ucv.ve; 4 Departamento de Imunologia, Instituto de Microbiologia Paulo de Góes, Universidade Federal do Rio de Janeiro, Rio de Janeiro 21941-592, Brazil; peralta@micro.ufrj.br

**Keywords:** *Schistosoma mansoni*, proteomic, *n*-butanol extract, antigens, host–parasite interactions

## Abstract

The *S. mansoni* adult worm *n*-butanol extract (Sm-AWBE) has been previously shown to contain specific *S. mansoni* antigens that have been used for immunodiagnosis of schistosomiasis in solid phase alkaline phosphatase immunoassay (APIA) and western blot (WB) analyses. Sm-AWBE was also used in immunoprotection studies against a fatal live-cercariae challenge in experimental mouse vaccination (~43% protection). The Sm-AWBE fraction was prepared by mixing adult worm membranous suspensions with aqueous-saturated *n*-butanol, centrifuging and recovering *n*-butanol-resistant proteins in the aqueous phase. Here we report a preliminary identification of Sm-AWBE protein components as revealed from a qualitative proteomic study after processing Sm-AWBE by 1D-gel electrophoresis, in-gel and in-solution tryptic digestions, and mass spectrometry analyses. We identified 33 proteins in Sm-AWBE, all previously known *S. mansoni* proteins and antigens; among them, immunomodulatory proteins and proteins mostly involved in host–parasite interactions. About 81.8% of the identified Sm-AWBE proteins are antigenic. STRING analysis showed a set of Sm-AWBE proteins configuring a small network of interactive proteins and a group of proteins without interactions. Functional groups of proteins included muscle contraction, antioxidant, GPI-anchored phosphoesterases, regulatory 14-3-3, various enzymes and stress proteins. The results widen the possibilities to design novel antigen combinations for better diagnostic and immunoprotective strategies for schistosomiasis control.

## 1. Introduction

The *S. mansoni* tegument, gut and extracellular vesicles are host–parasite interfaces where key parasite proteins can be targets for drug and immunological attacks [1], as well as representing candidates for *S. mansoni* diagnostic and vaccination [2]. Different from most classic parasite fractionation methods, for years we have used an organic solvent extraction with n-butanol of the adult female and male whole worm’s membranes fraction (containing tegumental, gut, and intracellular and vesicles parasite components, etc.), that we called *S. mansoni* adult worm butanol extract (Sm-AWBE).

By previously reported biochemical, electrophoretic, and western blot (WB) studies [3,4], we know that Sm-AWBE contains a mixture of enzymes, proteins and glycoproteins that have for long served as a source of very specific *S. mansoni* antigens for use in solid-phase alkaline phosphatase enzyme immunoassay (APIA) [3] and WB (4) diagnostic surveys in low endemic schistosomiasis regions of South America and the Caribbean [3,4,5,6]. The use of Sm-AWBE in both APIA and WB analyses increased the detection of positive cases even in communities with very low parasite loads, enhancing the epidemiological sensing [4].

Sm-AWBE contains molecules that have resisted denaturation to *n*-butanol [3,7]. Proteins resisting denaturation to organic solvents or detergents have particular molecular structures, 3D-conformations, or both, which make them insensitive to the action of those chemicals [8], such as proteins harboring long coil stretches (chaperones and stress proteins, etc.), highly glycosylated proteins (glycoproteins and proteoglycans) and GPI-anchored membrane proteins, etc. [9]. Typical *n*-butanol extracted proteins include GPI-anchored membrane parasite enzymes such as alkaline phosphatase (SmAP) [10,11,12].

On the other hand, proteases [13] are not usually found in Sm-AWBE, probably because they are inactivated, granting, by the way, Sm-AWBE to be a stable, protease-free, parasite extract. Seminal works using adult worm membrane extracts allowed us to describe the SmAP as a relevant parasite enzyme antigen localized on the *S. mansoni* tegumental surface and present in Sm-AWBE [3,14,15]. SmAP surface localization in adult worms as well as its expression across other *S. mansoni* life cycle stages was confirmed by other author studies [16]. Other antigenic enzymes were also detected in Sm-AWBE (including PDE and ATPase) [7]. However, anti-SmAP antibodies that originated during natural and experimental infections were only able to partially inhibit SmAP and SmPDE activities [7,17].

A majority of the Sm-AWBE components appear differently recognized when confronted to sera from *S. mansoni*-infected patients or experimentally infected animals in WB analyses [4]. WB detected not less than 20 antigenic components, in the molecular mass range of ~8 to >80 kDa, and various immunodominant bands in the 25 to 32 kDa interval, with recognition frequencies varying between 57.5 and 97.5%, were seen, depending on endemic region [4]. 

The high antigenicity and specificity bound to this extract prompted us to explore its immunoprotective capacity in experimental mouse immunizations; subcutaneous immunizations of mice with Sm-AWBE conferred a significant anti-*S. mansoni* protection level of about 43% relative to non-vaccinated animals [17]. Mice vaccinated in this way were also protected against a fatal challenge infection with living infective *S. mansoni* cercariae (500 cercariae) [17]. In this study, sera from vaccinated mice clearly recognized two Sm-AWBE antigens, actin and SmAP [17]. In other experiments, a WB analysis with sera from mice that were immunized and protected with irradiated cercariae showed an antigenic profile similar to those seen with sera from mice that were immunized and protected with Sm-AWBE [Sulbarán, G, unpublished results].

Multiple published genomic and proteomic studies have identified new antigens as well as druggable targets in classical soluble and membrane-bound *S. mansoni* fractions (tegument, gut, and excretions and secretions, etc.) [18,19,20,21,22,23,24,25,26,27]. The main aim of the present work was to have preliminary proteomics information on the identity of Sm-AWBE components. Here we report the identities of 33 different *S. mansoni* proteins present in the Sm-AWBE fraction, including enzymatic, regulatory, structural, anti-stress, secreted, transport, Ca^2+^-binding, inhibitory, immunomodulatory, tegumental, mitochondrial, and other proteins. About 81.8% of the 33 confirmed Sm-AWBE proteins are known *S. mansoni* antigens, most of them being relevant to host–parasite interactions. The importance of each molecule or a combination of them should be explored by functional and immunogenicity studies.

## 2. Results

### 2.1. Sm-AWBE Proteins Identified from the In-Gel and In-Solution Digestions

**Figure 1:**
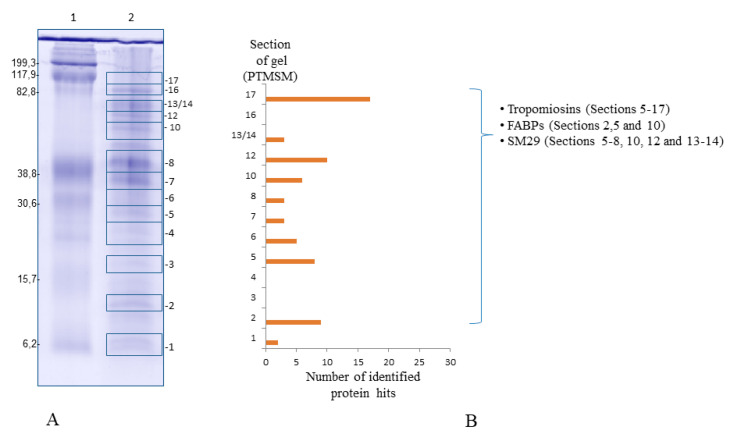
Sm-AWBE 1D gel electrophoresis separation and in-gel digestion proteomic analysis. (**A**): Sm-AWBE components separated by 12% SDS-PAGE under reduction conditions, Lane 1: Standard Molecular Weight, Lane 2: Different sections and fractions with proteins in the range of 7–100 kDa. (**B**): Representative chart showing the number of proteins identified by HPLC/MS-MS corresponding with each section or fraction of gel.

Several protein fragments and peptides were mass-detected more than once along the gel lanes in 1D Sm-AWBE gel electrophoresis separation (Figure 1A), in different molecular weight range sections (7–100 kDa range) (Figure 1A,B). In adult worms, it seems that we have various tropomyosin isoforms as well as several actins (Figure 1).

In-gel digested Sm-AWBE sample followed by HPLC/MS-MS analysis produced 66 protein hits (identified with at least two peptides) (Table S1); after data processing and refinement, 25 specific *S. mansoni* proteins were retained.

In-solution digested Sm-AWBE sample followed by HPLC/MS-MS analysis produced 139 protein hits; after data processing and refinement, 30 specific *S. mansoni* proteins (identified by at least two peptides) were retained (Table S2).

The total n° of proteins identified in Sm-AWBE after combining selected data from the in-gel (Table S1, n = 25) and the in-solution experiments (Table S2, n = 30), processing and refining, 33 specific *S. mansoni* proteins were finally retained as proteomics-identified Sm-AWBE components in this study (Table 1). Table S1 (in-gel) and Table S2 (in-solution) share 18 proteins with identical *Smp_number* (in **bold** in Table 1). 

The Sm-AWBE proteins reported in Table 1 appear all phenotypically distinct; this is confirmed in the brief descriptions, properties and functions given in Table S3. On the other hand, similarities in structural, biochemical, functional and immunological properties between some proteins can be found, which will allow us to classify and group them in some way. In Table 1 we can see, for instance: (1) predominance of enzymes (13/33 = 39.4%) with different catalytic activities (antioxidant, redox, transferase, phosphoesterase, aminopeptidase, hydratase, isomerase, GTPase and dehydrogenase); (2) relevant presence of regulatory proteins (12/33 = 36.4%) (anti-stress, chaperoning, folding, inhibitory, immunomodulatory, lectin-binding and stabilizing, etc.); and (3) remaining proteins (subcellular, extracellular and immunoactive, etc.).

### 2.2. Integrated Analysis of Sm-AWBE Proteins

An attempt was made to integrate relevant Sm-AWBE protein features in Table 2. In this Table, Sm-AWBE proteins were represented as points (●) and placed in columns corresponding to their biochemical or functional properties, or both. Smp_numbers were categorized in ascendant order in the first column on the left. Columns were consecutively ordered from outer-to-inner compartments of the worms⤙ body, and sub classified according to their physical and functional properties. Proteins placed in more than one column or class are endowed with more than one function, or otherwise, are exerting the same function in different locations. The total nº of proteins (●)/column/class is the result of a vertical counting (only one count per line even if repeated in the same line) and is given at the bottom of Table 2.

The adopted compartmental and/or functional classes were: (1) Extracellular (Secreted/exosomes/vesicles; Tegument/Surface, GPI-anchored, glycoproteins) (n = 23); (2) Signaling (Receptors, Transduction, Transmembrane/Membrane) (n = 10); (3) Intracellular (Cytoskeletal, Structural, Cytoplasmic, AWE (20) (n = 13); (4) Organelles (Endoplasmic Reticulum, Mitochondrion) (n = 6); (5) Transport/Motion (Ca^2+^-binding, Transport, Muscle) (n = 10); (6) Regulatory (Regulatory, Redox/Detoxifying, Chaperones/Scaffold, Immunomodulatory, Inhibitory) (n = 23); (7) Catalytic (n = 15) (Table 2). A host–parasite “Immunoactive” class was added at the right end of Table 2; proteins here included were *S. mansoni* allergens (All) [28], vaccine (Vac) target proteins [2,29], and literature-known *S. mansoni* antigens (Ags). All the Sm-AWBE proteins categorizing under “Immunoactive” (n = 27) represented 27/33 (81.8%) of the identified Sm-AWBE proteins.

To estimate the percent (%) of immunoactive proteins per class, we look at the horizontal proteins match between a given class and the “Immunoactive” class, related to total proteins in that class. Results were as follow: Extracellular 21/23 (91.3%), Signaling 6/10, Intracellular 11/13, Organelles 5/6, Transport/Motion 8/10, Regulatory 18/23 (78.3%), and Catalytic 12/15, the highest percent of immunoactive proteins accumulating in the “Extracellular” host–parasite interface (Table 2).

In Table 2, we see that most proteins are present in more than one class, some being present even in six classes, such as CALR (ER chaperone/lectin), PPIA, (PPIase/Cyclophilin), GST28mu (detoxifying enzyme) and PDI; that is, proteins mostly involved in protein–protein interactions and folding, etc., which are able to exert their functions in various compartments. Some of these proteins have been considered as targets for drugs, such as PPIA (CyP A), which is strongly inhibited by cyclosporin A—a schistosomicide [30], and vaccination (for instance, GST28mu) [31,32,33] (Table S3). Other proteins were found in five classes (ENO, SOD, and Actin, etc.), and so on, down to two classes. Proteins being present in one or two subcellular class may have unique functions and may represent unique targets.

### 2.3. STRING Analyses of Sm-AWBE Proteins 

**Figure 2:**
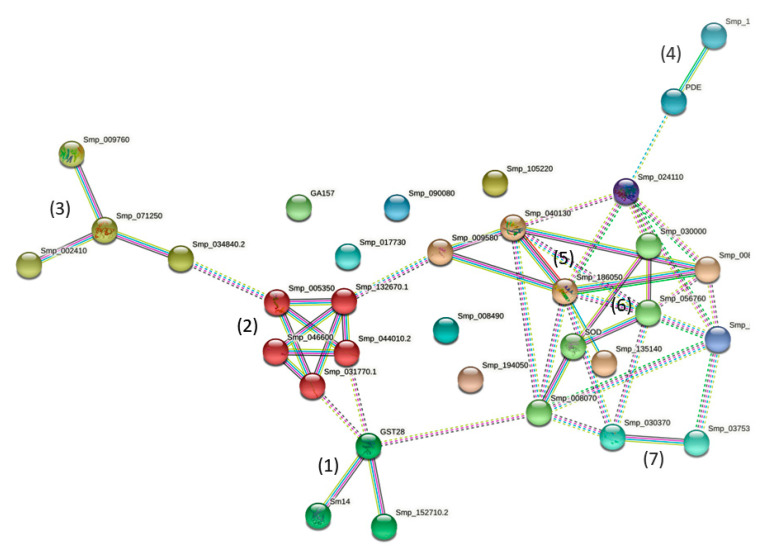
Sm-AWBE protein network (STRING analysis, https://string-db.org/, accessed on 9 December 2021). A cluster and protein groups appeared after applying the MLC inflation algorithm. (I) Proteins showing protein–protein interactions (PPIs): (**1**) Detoxifying transferases GST omega, GST28 mu, and FABP/Sm14 (fatty-acid binding protein); (**2**) Cluster with proteins involved in muscle contraction; (**3**) 14-3-3 regulatory proteins bound to rap1; (**4**) phosphoesterases; (**5**) chaperones/foldases; modulatory proteins; (**6**) antioxidant/redox proteins; (**7**) CALR, Trx-Mt. (II) Proteins without PPIs: Sm13/GA157, Sm200, serpin, DIF_5, ENO, Glycogenin-related, clfA, DLD.

Input of the 33 Sm-AWBE proteins into STRING (https://string-db.org/cgi/ accessed on 9 December 2021) resulted in a small network where 25/33 (75.8%) proteins exhibit protein–protein interactions (PPI+) whereas 8/33 (24.2%) did not establish protein interactions (PPI-) in that network, and remained single (Figure 2). Protein PPI+ or PPI- can be identified in Table 2.

Despite the fact that Sm-AWBE is not a classic subcellular fraction but just a chemically-extracted fraction, it was interesting to see how some Sm-AWBE proteins established interactions with other proteins in the network (Figure 2). When applying the MLC cluster inflation algorithm in the STRING menu, we can visualize one cluster and few interconnected protein groups (Figure 2).

(1) A three-protein group (Figure 2), configured with two detoxifying enzymes, GST omega (Smp_152710) [34] and GST28 mu (Smp_054160) [31,32,33,35,36], and the FABP (14 kDa fatty acid transporter) antigen [37,38]. These functional enzymes are well known proteins (Table S3) that have been used in anti-*S. mansoni* vaccination trials [2,39].

(2) A cluster with five tightly interconnected proteins, all involved in parasite’s muscle contraction: TPM-1 (Smp_044010) and TPM-2 (Smp_031770) (two IgE-inducers [40]), MCLR (Smp_132670) (a PZQ-target [41,42]), Ca^2+^-binding protein SM20 (Smp_005350) [43,44], and actin (Smp_046600) [29,41,45]; some of these proteins form myosin (the thick filaments structure in *S. mansoni* muscle) [46]. Applying the STRING analysis extensions (Pfam and SMART, etc.), we see that various of the above-mentioned Sm-AWBE proteins possess EF-hand domains, a stable structure capable of maintaining the ability to bind Ca^2+^ even under mildly denaturing conditions [47].

(3) A total of three 14-3-3 proteins (Smp_002410 epsilon 2, Smp_009760 protein homolog 1 and Smp_034840 protein homolog 2) [48,49,50] appears linked to the small GTPase rap1 (Smp_071250) (Figure 2) [51]. The 14-3-3 proteins have been tested in vaccination trials, producing 25-46% protection in terms of adult *S. mansoni* worm burden reduction [52] (Table S3).

(4) Phosphoesterases SmAP (Smp_155890) [3,5,11,14,15,16,53] and PDE (Smp_153390) [7,54,55] are key GPI-anchored tegumental enzyme antigens used in diagnostic (Table S3).

(5) Regulatory proteins UB (Smp_009580) [28,56,57] and PPIA (Smp_040130) [29,30] appeared linked to HSP70 (Smp_106930) [21,58] and HSP60 (Smp_008545) [59,60]. HSP60 and PPIA (CyP A) are mitochondrial proteins (UniProt). Interacting with HSP70, we found also the high voltage-activated calcium channel Cav1 [61].

(6) A small group of interacting proteins includes antioxidant and redox enzymes (Cu-Zn) SOD (Smp_176200) [41,62], Trx (Smp_008070) [63,64], PDI (Smp_056760) [65], as well as the modulatory LAP (Smp_030000) [66,67,68]. Trx and PDI have a thioredoxin domain.

(7) An isolated link was seen between CALR (Smp_030370) [69] and Mt TRx (Smp_037530) [70] (Figure 2).

Among the PPI- proteins we found the surface antigens Sm13 (Smp_195190) [71], GPI-anchored tegumental Sm200 (Smp_017730) [72,73,74], DIF_5 (CD59-like, SmLy6B) (Smp_105220) [75,76,77]; the serpin inhibitor (Smp_090080) [78,79]; Glycogenin-related protein (Smp_008490) [80,81], the clfA fibrinogen receptor A (Smp_194050) [82], ENO/phosphopyruvate hydratase (Smp_024110) [41,83,84,85,86], and mitochondrial DLD (Smp_046740) (Figure 2).

## 3. Discussion

Schistosomiasis continues to be a serious tropical disease and public health problem with a high level of morbidity in endemic countries [87]. Praziquantel (PZQ) is the only drug currently available for schistosomiasis treatment but it is unable to kill immature developing schistosomes, it does not prevent re-infection and its continued massive use conduces eventually to drug-resistant parasites. This persistent scenario maintains research interests for developing new drugs and anti-schistosome vaccines for prevention, control and elimination of schistosomiasis [88,89].

Experimental protection against schistosome infections was initially attempted using radiation-attenuated *S. mansoni* cercariae achieving 78% of immunoprotection level with one vaccination dose [90,91]. However, there was no progress with this line of research because of the problematical use of living attenuated infectious cercariae in humans, carrying too high a risk of side effects or of partially or unattenuated parasites becoming a patent infection [88]. Other strategies were then implemented, mostly using different parasite preparations from the *Schistosoma* life cycle stages, with different degrees of success [92], including secretory and antigenic molecules exhibiting specificity in host–parasite interactions [21].

More recently, multiple genomic and proteomic approaches have sought to identify new relevant antigens in different and diverse parasite preparations that could be used as antigens in an efficient schistosomiasis vaccine [1,12,18,19,20,21,22,23,24,25,26,27,93]. Various *S. mansoni* surface proteins identified by these authors in their parasite preparations have also been found previously by us in Sm-AWBE (alkaline phosphatase, phosphodiesterase 5, 200 kDa surface protein and actin) [7,17]. This and previous work confirm that Sm-AWBE contains vaccine antigens, and functional studies with identified putative antigens must be conducted to understand how to increase the Sm-AWBE vaccinating potential.

Floudas et al. [29] and Samoil et al. [23] have analyzed the content of exosome-like vesicles derived from *S. mansoni* [23,29] (Table 2). Among the identified Sm-AWBE proteins (Table 1), about 85.1% can be assigned to the extracellular and secreted vesicles and the parasite surface (host–parasite interface) compartment, and about 91.3% of these proteins may be immunoactive (Table 2). Among the 130 *S. mansoni* proteins identified in exosome vesicles by Samoil et al. [23], at least 14 were also found in Sm-AWBE (14-3-3 epsilon, Glycogenin-related, Ubiquitin, 14-3-3 protein homolog 1, Sm200, Enolase, LAP, PPIA, Actin-2, GST28, Rap1, Sm32, FABP and HSP70). Some of the surface proteins identified by Braschi et al. [73] and Fonseca et al. [2] were also found in Sm-AWBE (namely, phosphodiesterase 5, alkaline phosphatase, 200 kDa surface protein and actins). Among the 43 protein spots identified through a proteomic work in an adult worm antigenic extract (AWE) of our *S. mansoni* JL strain [20], eight (24.2%) were present in Sm-AWBE (Table 2). Among the *S. mansoni* proteins described by Curwen et al. [94] in a comparative proteomics study between extracts from Praziquantel (PZQ)-resistance vs. (PZQ)-susceptible worms, 14-3-3 protein, HSP70, GST28, ENO and actin were found in AWE [20] and Sm-AWBE (Table 2).

Ludolf et al. [41] evaluated the immunoreactivity of various *S. mansoni* proteins against pooled *S. mansoni*-infected sera; in their work, they found 47 adult and egg antigens recognized by those sera. We identified at least six of those proteins in Sm-AWBE: 14-3-3 epsilon isoform (Smp_034840), ENO/Phosphopyruvate hydratase) (Smp_024110), actin (Smp_046600), FABP/Sm14 (Smp_095360), GST28mu (Smp_054160), HSP70 (Smp_106930) and SOD (Cu/Zn) (Smp_176200). All the above results support the validity of Sm-AWBE as an antigen-rich fraction. Crosnier et al. [89] made a systematic screening of 96 *S. mansoni* cell-surface and secreted antigens, and they concluded not to have identified any strongly protective vaccine candidates among these proteins in a mouse model of infection. Some antigens reported by these authors are present in Sm-AWBE, namely: (1) Surface GPI-anchored Sm200 (Smp_017730), DIF_5 (SmLyB, Cd59.2) (Smp_105220) and Sm13 (Smp_195190). (2) Secreted NPP-5/PDE (Smp_153390). It would be interesting to explore, however, if these “weakly” protective antigens could be suitable for diagnosis [93].

An overview of the Sm-AWBE proteins identified by proteomics in the present study reveals that Sm-AWBE contains a heterogeneous mixture of many different types of proteins (Table 1 and Table 2), all having in common that they are resistant to *n*-butanol denaturation. It has been described that the action of this organic solvent on biological material is closely associated to its toxicity effects. This varies across different solvents and is assumed to be closely related to their hydrophobicity (logP). Organic solvents with logP values between one and five were found to be particularly toxic due to their similar hydrophobicity to membrane and easy penetration into membrane, especially butanol (0.88, logP) [95], which is ranked as one of the most toxic organic solvents. Regarding living organisms, it is worth mentioning that *E. coli* exposed to butanol undergo several diverse stress conditions, including oxidative stress, acid stress, heat shock and envelope stress [96,97]. This indicates that living organisms would respond to the alcohol aggression by producing anti-stress proteins, themselves expected to be resistant to the denaturing agent. Complementary to this, proteins that are essential for organism survival in hostile environments, as the blood system for a pathogen, should endure denaturation when the forces that maintain the secondary, tertiary and quaternary structures of proteins could be disrupted. Differences among *Schistosoma* species are related to proteins involved in response to stress (changes in pH, attack by ROS metabolites, depletion of essential nutrients and heat shock proteins, etc.); differences in stress-inducible HSP70 between two schistosome or parasite species may be related to differences in host-related stresses [21]. Antioxidant enzymes (Trx, SOD and GST) have been shown to play important roles in the protection of *S. mansoni* in the host–parasite interplay [64,70,98,99,100]. The presence of antioxidant and redox enzymes in Sm-AWBE obeys to being oxidative stress-resistant and at the same time confers protein stability against denaturizing agents such as, in this case, *n*-butanol. Other resistant proteins in Sm-AWBE are GPI-anchored glycoproteins, hydrophobic proteins, protein with predominance of α-helices, anti-parallel β sheets, EF-hand, Trx and disordered domains, etc. Membrane proteins that keep membrane fluidity [101], in particular, chaperones and folding proteins (PPIases), are also associated with tolerance to organic solvent [102]. It is noteworthy that the most abundant proteins found in *S. mekongi* egg proteome were antioxidant proteins [25].

When Sm-AWBE proteins were input into STRING for analysis, we saw a small network with 25 proteins exhibiting PPIs (PPI+) and eight proteins with no protein interaction (PPI-). Among proteins establishing PPIs, we found one cluster and various small groups of interacting proteins. Cluster #2 was formed by tightly interacting worm muscle proteins, which may be of relevance for drugs targeting muscle contraction (PZQ targets). This cluster contains the proteins that ensemble the myosin thick filament. *S. mansoni* has a complex muscle tissue, a hybrid between striated and smooth muscle [46], which allows the parasite to attach to the mesenteric veins but also to migrate, helping the parasite to co-exist inside the human host. It has been suggested that the regulatory myosin chain is a target for PZQ [42]. The main consequence of PZQ action is a sustained hypercontraction of the *Schistosoma* muscle that derives in tetanus and detachment from the tegument [103]. Actins are, however, more abundantly present in adult worm extracts (AWE, obtained by homogeneization and centrifugation at 12,000× *g*/2 h/4 °C) [20] than Sm-AWBE. Adult *S. mansoni* male worms have tegumental surface spines composed of crystallized actin [45]. It has been hypothesized that some actin epitopes could be exposed during the course of infection and in fact anti-*S. mansoni* actin immune responses have been found in individuals living in endemic zones and in mice experimentally immunoprotected with Sm-AWBE [17].

It is difficult to extend functional interpretations of protein–protein interactions to any group of proteins in this Sm-AWBE network, except for some structural and functional similarities, because proteins in Sm-AWBE do not correspond to a functional sub proteome.

Among proteins without PPIs, ENO is a relevant glycolytic enzyme that also activates plasminogen, and it is involved in the processes of infection and migration of the parasite, reducing the host immune function as well as preventing the immune attack of the host to the parasites [83]. It has been suggested that ENO may have also a relationship with susceptibility and resistance to PZQ [27].

To summarize, in Sm-AWBE we have proteins resistant to harsh treatments, such as stress proteins, chaperones, antioxidant and redox proteins, and various other proteins probably counteracting host responses in living worms. Proteins that should be expected to be naturally resistant to aggressive external factors are proteins such as secreted proteins, proteins neutralizing free radicals, to assist or to repair unfolded proteins, to repair DNA damages, to evade innate host immune attacks (DIF_5 and LAP, etc.), and even proteins able to modulate the host immune responses, such as those described by [41]. We have extrapolated the presence of 27/33 (81.8%) different putative antigens in Sm-AWBE (Table 2), some of them probably good for immunoprotection [17], some probably better for diagnosis [3,4]. When dissecting *S. mansoni* antigens for diagnosis, vaccination or immunity, different strategies may be required, particularly in endemic regions, depending on if we want just diagnosis, to induce protection against the infection or to develop resistance to reinfection. Resistance to reinfection is usually considered important in endemic regions that have an ongoing low-level parasite transmission. Resistance to reinfection is usually controlled by IgE responses [35,94,104], and we have signaled the putative presence of IgE-inducing antigens in Sm-AWBE (Table 2) [17]. We have also acknowledged the presence in Sm-AWBE of eight proteins identified by other authors as immunomodulatory and/or involved in the evasion of the host immune responses (UB, ENO, PPIA, serpin, DIF_5, HSP70, SmAP, cflA) (Table 2), which is an important issue to take into account when making vaccines with high percent of efficacy. 

## 4. Conclusions

The *n*-butanol extraction procedure is an old but very simple technique that we have used since 1974 to “solubilize” membrane-bound surface enzymes such as the *S. mansoni* alkaline phosphatase [3,4,14,17]. This procedure has been used in other biological systems as a method for the extraction of human organ-specific neoantigens from cancer cells and plasma membranes (immunoprotective tumor antigens) [105], for the selective extraction of surface glycoprotein antigens from human melanoma cells [106], and for the extraction of antioxidant and anticancer compound activities from plants [107], among other examples. When applied to *S. mansoni* membranes, it produces a parasite fraction that the present proteomic work has revealed to be rich in denaturizing-resistant proteins (most of them antigenic and some of them drug targetable) of particular functional and immunological relevance, and playing fundamental roles in the host–parasite interactions.

The presence of heterogeneous, specific *S. mansoni* antigens in Sm-AWBE strongly confirm the usefulness of this fraction in WB analyses in low transmission areas because of its extreme sensitivity for schistosomiasis mansoni diagnosis [4]. The present results emphasize that *n*-butanol extraction combined with proteomic identification is a useful tool to dissect and explore complex molecular systems in host–pathogen interactions. Quantitative proteomic studies will assess relative protein abundance in the Sm-AWBE fraction. We should now envisage how to implement the newly acquired information to suggest interesting drug targets, to recommend adequate *S. mansoni* antigens (or recombinants) for immune protective trials, all aiming at a better control of schistosomiasis. Finally, we think that a major contribution of the present results is to realize that Sm-AWBE gathered in one fraction key proteins that are involved in host–parasite interactions, being proteins that are amazingly resistant to structural disruption and denaturation, underlying their critical role for the parasite defense and survival face to host attacks and hostile microenvironments.

## 5. Materials and Methods

### 5.1. Obtention of Adult S. mansoni Worms

Adult *S. mansoni* (Venezuelan JL strain) worms were obtained by perfusion of hamsters infected with 400 cercariae 7 weeks before, washed in sterile saline, and frozen at −80 °C until used [14,108].

### 5.2. Adult S. mansoni Worms n-Butanol Extract (Sm-AWBE) Preparation

Worms (male and females) were homogenized in Potter-Elvejhem at 4 °C with 50 mM Tris-HCl pH 8.0 (Tris-HCl buffer). The resulting homogenate (containing heterogeneous soluble and subcellular particles such as nuclei, tegument fragments, muscle debris, parenchyma, vesicles, discoid bodies, microsomes, ER fragments and mitochondria, etc.) was ultracentrifuged for one (1) hour at 100,000× *g*; the supernatant (soluble fraction) was discarded, and the membranous pellet suspended by homogenization in the same buffer volume and spun for a 2nd time under the same conditions. The buffer-washed particulate membranous sediment was conveniently suspended in 50 mM Tris/HCl buffer pH 8.0 and water-saturated *n*-butanol (90.1% *n*-butanol, 9.9% water) added in a vol/vol ratio. This suspension was vigorously stirred for 15 min to force partition of hydrophilic and hydrophobic components into the aqueous and the organic phases, respectively. The resulting emulsion was centrifuged at 20,000× *g* at 4 °C for 5 min, the lower aqueous phase (named adult worm butanol extract or AWBE) was recovered, the upper lipid organic phase removed and the solid material at the interface re-extracted as above with water-saturated *n*-butanol. The aqueous fractions (AWBEs) were combined and residual *n*-butanol removed from AWBE by overnight dialysis against the same buffer at 4 °C. After dialysis, “extracted” membrane components tend to precipitate and it is necessary to add Triton X-100 at a final 0.1% (*v*/*v*) concentration to keep them in solution. The resulting extract is a soluble and filterable fraction enriched in glycosylated and resistant-to-denaturation proteins, being a “standard” worm preparation not changing in composition over time, which can be conserved at room temperature and reused after filtering as needed, which is very useful when used as source of antigens in diagnosis field works [3,4,17]. Protein concentration was determined using bovine serum albumin as the standard protein according to the method of Bradford [109].

### 5.3. One-Dimensional Gel Electrophoresis Separation and In-Gel Protein Digestion of Sm-AWBE

A total of fifty micrograms of Sm-AWBE proteins were diluted in sample buffer (50 mM Tris-Cl, pH 6.8, 100 mM β mercaptoethanol, 2% SDS, 0.1% bromophenol blue, 10% glycerol) and heated at 100 °C for 5 min. After this step, the proteins were separated by 1D-SDS-PAGE 12% [110] using a Mini Protean II Dual Slab Cell, Bio Rad at a constant voltage of 120 V. Protein bands were visualized by Coomassie blue G-250 staining and sliced horizontally in sections of gel pieces to perform the in-gel digestion. Briefly, visible bands were cut, picked up and reduced with 50 mM DDT and alkylated using 100 mM of iodocetamide. The proteins were trypsin-digested with 25 μL of enzyme (80 ng/mL in 25 mM NH_4_HCO_3_, pH 7.8) (MS grade, Promega, Maddison, WI, USA) per piece of gel at 37 °C for 15 h. After the gel extraction step, the resultant peptide mixture was loaded onto a ZipTip C-18 microcolumn (Millipore, Billerica, MA, USA), dried and finally resuspended in a solution containing 0.1% formic acid in 2% acetonitrile (ACN) for mass spectrometry analysis.

### 5.4. In-Solution Digestion of Sm-AWBE Proteins

A total of fifty micrograms of extract was precipitated with 10% TCA, washed as described above and resuspended in 20 μL of 25 mM NH_4_HCO_3_. The digestion of proteins in solution was carried out with 500 ng of MS-grade Trypsin (Promega) in 25 mM NH_4_HCO_3_ at 37 °C for 14 h. The cleaning, elution and preparation of the peptide solution for mass spectrometry analysis were also performed as described above.

### 5.5. Mass Spectrometry

A total of two microliters of cleaned peptide obtained by in-solution digestion (500 ng of digested protein) and 7–10 µL obtained by in-gel digestion were injected at 4 °C for the nano-LC-based separation (partial loop) combined with mass spectrometry analysis on an LC-ESI-Q-TOF micromass instrument (Waters Co., Williford, AR, USA) with data-dependent acquisition (DDA). The peptide chromatography separation was performed in a nano-ACQUITY system equipped with a Symmetry C18 5-μm diameter, 5 × 300 precolumn (trapping flow 200 nL/min and run time trap 10 min) and an Atlantis 100 × 100, 1.7 μm diameter analytical reversed-phase C18 column with a solution gradient of 5–50% mobile phase (water (Solution A) and acetonitrile (Solution B) over 50 min at flow rate of 350 nL/min (Time(min)/min/%B solution Curve–1. Initial/2%B, 2. 5 min/10 %B, 3. 30 min/30%B, 4. 50min/50%B). The column temperature was maintained at 35 °C and the lock mass used was phosphoric acid, delivered by an auxiliary pump at a flow rate of 200 nL/min. The conditions for peptide ionization included a source temperature of 80 °C, capillary voltage of 3500 V, positive polarity, and a sample cone voltage of 35 V. Mass spectra were acquired with the TOF mass analyzer operating in the V-mode, and spectra were integrated over 1 s of scanning and with 0.1-s interscan intervals. The MS/MS mass spectra were acquired at a m/z range of 50 to 1700. The three most intense ions (top 3) were selected on MS scan using the reference mass acquired and the continuous fragmentation were performed in continuous fragmentation mode in 10 eV collision energy.

### 5.6. Data Analysis and Protein Identification

The raw data obtained by DDA were then processed using the ProteinLynx 2.5 software (Waters MS Technologies, Manchester, UK). Peak list files were used to search and to identify proteins by use of Peaks X+ software (Bioinformatic Solutions, Waterloo, Canada), matched against a *Schistosoma mansoni* database protein UNIPROTDB (access: July 2020). The search parameters were set as follows: two missed cleavage, carbamidomethyl (C) as a fixed modification, oxidation (M) as a variable modification, 0.1 of MS tolerance, 0.1 of MS/MS tolerance, +2 +3 and +4 charges and False Discovery Rate (<1). All the identifications were checked manually and only considered as valid proteins with 2 or more peptides. Specific *S. mansoni* proteins in Sm-AWBE were further analyzed through STRING (https://string-db.org, accessed on 9 December 2021) and UNIPROT (https://www.uniprot.org/uniprot/, accessed on 9 December 2021) for additional properties and protein–protein interactions (PPIs) between components, also checked by *S. mansoni* database (https://parasite.wormbase.org/Schistosoma, accessed on 12 March 2020) and a gene expression Atlas at http://schisto.xyz/geneexp/, accessed on 12 March 2020.

## Figures and Tables

**Table 1:** Sm-AWBE proteins identified by proteomics in-gel and in-solution (n = 33).

Accession	Description (Short Names)
Smp_002410	14-3-3 epsilon 2
**Smp_005350**	**20 kDa calcium**-**binding protein** (**SM20**)
**Smp_008070**	**Thioredoxin** (**Trx**)
**Smp_008490**	**Glycogenin**-**related**
**Smp_008545**	**Heat shock protein 60** (**HSP60**)
Smp_009580	Ubiquitin (UB)
Smp_009760	14-3-3 protein homolog 1
Smp_017730	200 kDa GPI-anchored surface protein (Sm200)
**Smp_024110**	**Enolase**/**Phosphopyruvate hydratase** (**ENO**)
**Smp_030000**	**Leucine aminopeptidase** (**M17**)/**putative cytosol aminopeptidase** (**LAP**)
**Smp_030370**	**Calreticulin** (**CALR**)
**Smp_031770**	**Tropomyosin**-**2** (**TPM**-**2**)
Smp_034840	14-3-3 protein homolog 2 (14-3-3 protein epsilon)
Smp_037530	Mitochondrial thioredoxin (Mt Trx)
Smp_040130	Peptidyl-prolyl *cis*-*trans* isomerase (CyP A) (PPIA)
**Smp_044010**	**Tropomyosin**-**1** (**TPM**-**1**)
**Smp_046600**	**Actin** (**s**)
Smp_046740	Dihydrolipoyl dehydrogenase (DLD)
**Smp_054160**	**Glutathione S**-**transferase class**-**mu 28 kDa isozyme** (**GST28**)
**Smp_056760**	**Protein disulfide**-**isomerase** (**PDI**)
Smp_071250	Putative rap1 and (Rap1)
Smp_090080	Serpin, putative
**Smp_095360**	**14 kDa fatty acid**-**binding protein** (**FABP**/**Sm14**)
**Smp_105220**	**DIF_5** (**CD59**-**like, SmLy6B**)
Smp_106930	Heat shock 70 kDa protein homolog (HSP70)
Smp_132670	Myosin regulatory light chain, putative (MRLC)
Smp_135140	High voltage-activated calcium channel Cav1
Smp_152710	Glutathione-S-transferase omega, putative (GST omega)
**Smp_153390**	**Nucleotide pyrophosphatase**/**phosphodiesterase 5** (**NPP**-**5**)/**Ecto**-**phosphodiesterase** (**PDE**)
**Smp_155890**	**Alkaline phosphatase** (**AP**)
**Smp_176200**	**Superoxide dismutase** (**Cu**-**Zn**) (**SOD Cu**-**Zn**)
Smp_194050	Clumping factor A (Fibrinogen-binding protein A) (Fibrinogen receptor A), putative (clfA)
**Smp_195190**	**13 kDa tegumental antigen Sm13**/**GA157** (**Sm13**)

Data were extrapolated from Tables S1 and S2. In **bold**: Proteins appearing identical in the in-gel and the in-solution experiments (n = 18).

**Table 2:** Integration of Sm-AWBE protein components according to their properties and functions.

			Extracellular		Signaling			Intracellular				Organelles		Transport/Motion			Regulatory					Cat.	Immunoactive			
Accession	Proteins Short Names	Av. Mass	Scr	TgS	Rcp	Trd	TrM	Cyk	Str	Cyt	AWE	ER	Mit	Ca-b	Trp	Msl	Reg	RxD	Ch/F	Imd	Inh	Cat.	All	Vac	Ags	PPIs
Smp_002410	14-3-3 epsilon 2	28432	●			●	●										●							●	●	+
Smp_005350	**SM20**	57939		●										●									●		●	+
Smp_008070	**Trx**	11924																●				●	●	●	●	+
Smp_008490	**Glycogenin-related**	36511	●				●															●				-
Smp_008545	**HSP60**	59554											●						●				●		●	+
Smp_009580	UB	18327	●							●										●		●	●			+
Smp_009760	14-3-3 protein homolog 1	28371	●			●	●										●								●	+
Smp_017730	Sm200	186533	●	●																				●	●	+
Smp_024110	**ENO**	47006	●	●						●	●									●		●			●	+
Smp_030000	**LAP**	55676	●	●						●												●	●		●	+
Smp_030370	**CALR**	45377	●							●		●		●					●						●	+
Smp_031770	**TPM-2**	32696														●	●								●	+
Smp_034840	14-3-3 protein epsilon	28754	●			●	●										●								●	+
Smp_037530	Mt Trx	12424											●					●				●				+
Smp_040130	PPIA (CyP A)	17671	●	●									●			●	●		●	●		●	●	●	●	+
Smp_044010	**TPM-1**	32954														●	●						●		●	+
Smp_046600	**Actin** (**s**)	41731	●	●				●	●	●	●					●						●			●	+
Smp_046740	DLD	52868											●					●				●	●			+
Smp_054160	**GST28**	23819	●	●							●				●			●				●		●	●	+
Smp_056760	**PDI**	54160		●							●	●						●				●			●	+
Smp_071250	Rap1	20700	●			●	●										●									+
Smp_090080	Serpin, putative	46005	●								●									●	●		●		●	-
Smp_095360	**FABP**/**Sm14**	11923	●							●					●	●								●	●	+
Smp_105220	**DIF_5**	14191		●																●			●	●	●	-
Smp_106930	HSP70	69859	●	●							●								●	●					●	+
Smp_132670	MRLC	22719												●		●									●	+
Smp_135140	Cav1	87668					●		●					●												-
Smp_152710	GST omega	27532									●							●				●				+
Smp_153390	**NPP-5**/**PDE**	51434	●	●			●															●		●	●	+
Smp_155890	AP	46318		●			●													●		●			●	+
Smp_176200	SOD Cu-Zn	15910	●							●	●							●				●	●	●	●	+
Smp_194050	clfA	43482			●															●						-
Smp_195190	**Sm13**/**GA157**	11922	●	●			●																		●	-
**n = 33**			**Scr**	**TgS**	**Rcp**	**Trd**	**TrM**	**Cyk**	**Str**	**Cyt**	**AWE**	**ER**	**Mit**	**Ca-b**	**Trp**	**Msl**	**Reg**	**RxD**	**Ch/F**	**Imd**	**Inh**	**Cat.**	**All**	**Vac**	**Ags**	
			**23**		**10**			**13**				**6**		**10**			**23**					**15**	**27**			

In **bold**: Sm-AWBE proteins detected in both in-gel and in-solution experiments (n = 18). Sm-AWBE proteins were represented by dots [●] and dots placed in the columns according to their biochemical and/or functional properties. **Abbreviations: Extracellular** (**Scr** = Secreted/Vesicles; **TgS** = Tegument/Surface (including Glycoproteins and GPI-anchored proteins)); **Signaling** (**Rcp** = Receptor; **Trd** = Transducing; **TrM** = transmembrane, plasma membrane, integral membrane protein, membrane-associated); **Intracellular** (**Cyk** = Cytoskeleton; **Str** = Structural/Scaffold; **Cyt** = Cytosolic; **AWE** = Soluble « Adut Worm Extract »); **Organelles** (**ER** = Endoplasmic Reticulum; **Mit** = Mitochondrion); **Transport**/**Motion** (**Ca-b** = Calcium-binding; **Trp** = Transport; **Msl** = Muscle); **Regulatory** (**Reg** = Regulatory; **RxD** = Redox/Detoxyfying; **Ch**/**F** = Chaperone/Folding; **Imd** = Immunomodulatory; **Inh** = Inhibitors); **Catalytic** (**Cat.** = Enzymes/Metabolism); **Immunoactive** (**All** = Allergens; **Vac** = Vaccinating; **Ags =** Antigens).

## Data Availability

Not applicable.

## Supplementary Materials

The following are available online at https://www.mdpi.com/article/10.3390/pathogens11010022/s1, Table S1: Sm-AWBE proteins identification after 1D-SDS/PAGE, bands gel digestion, MS Analysis, Table S2: Proteins identification after in-solution digestion of Sm-AWBE, Table S3: Known molecular and functional properties of the proteomics-identified Sm-AWBE proteins.

## References

[1] Sotillo J., Pearson M.S., Becker L., Mulvenna J., Loukas A. (2015). A quantitative proteomic analysis of the tegumental proteins from *Schistosoma mansoni* schistosomula reveals novel potential therapeutic targets. Int. J. Parasitol..

[2] Fonseca C.T., Figueiredo Carvalho G.B., Alves C.C., Melo T.T. (2012). *Schistosoma* tegument proteins in vaccine and diagnosis development: An update. J. Parasitol. Res..

[3] Pujol F.H., Alarcón de Noya B., Cesari I.M. (1989). Immunodiagnosis of schistosomiasis mansoni with APIA (alkaline phosphatase immunoassay). Immunol. Investig..

[4] Cesari I.M., Ballen D.E., Mendoza L., Matos C. (2005). Detection of *Schistosoma mansoni* membrane antigens by immunoblot analysis of sera of patients from low-transmission areas. Clin. Diagn. Lab. Immunol..

[5] Cesari I.M., Ballén D.E., Mendoza L., Ferrer A., Pointier J.P., Kombila M., Richard-Lenoble D., Théron A. (2014). Comparative evaluation of *Schistosoma mansoni, Schistosoma intercalatum*, and *Schistosoma haematobium* alkaline phosphatase antigenicity by the alkaline phosphatase immunoassay (APIA). Parasitol. Res..

[6] Alarcón de Noya B., Cesari I.M., Losada S., Colmenares S., Balzán C., Hoebeke J., Noya O. (1997). Evaluation of alkaline phosphatase immunoassay and comparison with other diagnostic methods in areas of low transmission of schistosomiasis. Acta Trop..

[7] Cesari I.M., Bouty I., Bout D.B., de Noya B.A., Hoebeke J. (1992). Parasite enzymes as a tool to investigate immune responses. Mem. Inst. Oswaldo Cruz.

[8] Stepankova V., Bidmanova S., Koudelakova T., Prokop Z., Chaloupkova R., Damborsky J. (2013). Strategies for stabilization of enzymes in organic solvents. ACS Catal..

[9] Solá R.J., Griebenow K. (2009). Effects of glycosylation on the stability of protein pharmaceuticals. J. Pharm. Sci..

[10] Pearce E.J., Sher A.J. (1989). Three major surface antigens of *Schistosoma mansoni* are linked to the membrane by glycosylphosphatidylinositol. Immunology.

[11] Pujol F.H., Cesari I.M. (1990). Antigenicity of adult alkaline phosphatase. Parasite Immunol..

[12] Bhardwaj R., Skelly P.J. (2011). Characterization of Schistosome tegumental alkaline phosphatase (SmAP). PLoS Negl. Trop. Dis..

[13] Caffrey C.R., McKerrow J.H., Salter J.P., Sajid M. (2004). Blood ‘n’ guts: An update on schistosome digestive peptidases. Trends Parasitol..

[14] Cesari I.M. (1974). *Schistosoma mansoni*: Distribution and characteristics of alkaline and acid phosphatase. Exp. Parasitol..

[15] Cesari I.M., Simpson A.J.H., Evans W.H. (1981). Properties of a series of tegumental membrane bound phosphohydrolase activities of *Schistosoma mansoni*. Biochem. J..

[16] Araujo-Montoya B.O., Rofatto H.K., Tararama C.A., Farias L.P., Oliveira K.C., Verjovski-Almeida S., Wilson R.A., Leite L.C.C. (2011). *Schistosoma mansoni*: Molecular characterization of alkaline phosphatase and expression patterns across life cycle stages. Exp. Parasitol..

[17] Sulbarán G., Noya O., Brito B., Ballén D.E., Cesari I.M. (2013). Immunoprotection of mice against *Schistosomiasis mansoni* using solubilized membrane antigens. PLoS Negl. Trop. Dis..

[18] Wilson R.A., Curwen R.S., Braschi S., Hall S.L., Coulson P.S., Ashton P.D. (2004). From genomes to vaccines via the proteome. Mem. Inst. Oswaldo Cruz..

[19] Hansell E., Braschi S., Medzihradszky K.F., Sajid M., Debnath M., Ingram J., Lim K.C., McKerrow J.H. (2008). Proteomic analysis of skin invasion by blood fluke larvae. PLoS Negl. Trop. Dis..

[20] Losada S., Sabatier L., Hammann P., Lemaitre-Guillier C., Matos C., Bermúdez H., Lorenzo M., Noya O. (2011). A combined proteomic and immunologic approach for the analysis of *Schistosoma mansoni* cercariae and adult worm protein extracts and the detection of one of the vaccine candidates, Sm28GST, from a Venezuelan parasite isolate. Investig. Clin..

[21] Mutapi F. (2012). Helminth parasite proteomics: From experimental models to human infections. Parasitology.

[22] Nahum L.A., Mourão M.M., Oliveria G. (2012). New frontiers in schistosoma genomics and transcriptomic. J. Parasitol. Res..

[23] Carvalho G.B.F., Resende D.M., Siqueira L.M.V., Lopes M.D., Lopes D.O., Coelho P.M.Z., Texeira-Carvalho A., Ruiz J., Fonseca C.T. (2017). Selecting targets for the diagnosis of *Schistosoma mansoni* infection: An integrative approach using multi-omic and immunoinformatics data. PLoS ONE.

[24] Samoil V., Dagenais M., Ganapathy V., Aldridge J., Glebov A., Jardim A., Ribeiro P. (2018). Vesicle-based secretion in schistosomes: Analysis of protein and microRNA (miRNA) content of exosome-like vesicles derived from *Schistosoma mansoni*. Sci. Rep..

[25] Thiangtrongjit T., Adisakwattanab P., Limpanontc Y., Dekumyoyb P., Nuamtanongb S., Chusongsang P., Chusongsang Y., Reamtong O. (2018). Proteomic and immunomic analysis of *Schistosoma mekongi* egg proteins. Exp. Parasitol..

[26] Carson J.P., Robinson M.W., Hsieh M.H., Cody J., Le L., You H., McManus D.P., Gobert G.N. (2020). A comparative proteomics analysis of the egg secretions of three major schistosome species. Mol. Biochem. Parasitol..

[27] Afonso A.J., Pinto Fonseca S., Pinto-Almeida A., Mendes T., Ferreira P., Belo S., Anibal F., Allegretti S., Galinaro C.A., Carrilho E. (2021). Comparative proteomics reveals characteristic proteins on praziquantel-resistance in *Schistosoma mansoni*. Dryad.

[28] Farnell E.J., Tyagi N., Ryan S., Chalmers I.W., Pinot de Moira A., Jones F.M., Wawrzyniak J., Fitzsimmons C.M., Tukahebwa E.M., Furnham N. (2015). Known allergen structures predict *Schistosoma mansoni* IgE-binding antigens in human infection. Front. Immunol..

[29] Floudas A., Cluxton C.D., Fahel J., Khan A.R., Saunders S.P., Amu S., Alcami A., Fallon P.G. (2017). Composition of the *Schistosoma mansoni* worm secretome: Identification of immune modulatory Cyclophilin, A. PLoS Negl. Trop. Dis..

[30] Bout D., Deslée D., Capron A. (1986). Antischistosomal effect of cyclosporin A: Cure and prevention of mouse and rat schistosomiasis mansoni. Infect. Immun..

[31] Balloul J.M., Boulanger D., Sondermeyer P., Dreyer D., Capron M., Grzych J.M., Pierce R., Carvallo D., Lecocq J.P., Capron A. (1987). Vaccination of baboons with a P28 antigen of *Schistosoma mansoni* expressed in *Escherichia coli*. Molecular Paradigms for Eradicating Helminthic Parasites.

[32] Wolowczuk I., Auriault C., Bossus M., Boulanger D., Gras-Masse H., Mazingue C., Pierce R., Grezel D., Reid G.D., Tartar A. (1991). Antigenicity and immunogenicity of a multiple peptidic construction of the *Schistosoma mansoni* Sm-28 GST antigen in rat, mouse, and monkey. 1. Partial protection of Fischer rat after active immunization. J. Immunol..

[33] Riveau G., Poulain-Godefroy O., Dupré L., Remoué F., Mielcarek N., Locht C., Capron A. (1998). Glutathione S-Transferases of 28kDa as major vaccine candidates against Schistosomiasis. Mem. Inst. Oswaldo Cruz..

[34] Girardini J., Amirante A., Zemzoumi K., Serra E. (2002). Characterization of an omega-class glutathione S-transferase from *Schistosoma mansoni* with glutaredoxin-like dehydroascorbate reductase and thiol transferase activities. Eur. J. Biochem..

[35] Torres-Rivera A., Landa A. (2008). Glutathione transferases from parasites: A biochemical view. Acta Trop..

[36] Driss V., El Nady M., Delbeke M., Rousseaux C., Dubuquoy C., Sarazin A., Gatault S., Dendooven A., Riveau G., Colombel J.F. (2016). The schistosome glutathione S-transferase P28GST, a unique helminth protein, prevents intestinal inflammation in experimental colitis through a Th2-type response with mucosal eosinophils. Mucosal Immunol..

[37] Moser D., Tendler M., Griffiths G., Klinkert M.Q. (1991). A 14-kDa *Schistosoma mansoni* polypeptide is homologous to a gene family of fatty acid binding proteins. J. Biol. Chem..

[38] Tendler M., Almeida M.S., Vilar M.M., Pinto P.M., Limaverde-Sousa G. (2018). Current status of the Sm14/GLA-SE schistosomiasis vaccine: Overcoming barriers and paradigms towards the first anti-parasitic human(itarian) vaccine. Trop. Med. Infect. Dis..

[39] Eyayu T., Zeleke A.J., Worku L. (2020). Current status and future prospects of protein vaccine candidates against *Schistosoma mansoni* infection. Parasite Epidemiol. Control..

[40] Silas S., Fitzsimmons C.M., Jones F.M., Pinto de Moira A., Wawrzyuniak J., Tukahebwa E.M., Dunne D.W. (2014). Human IgE responses to different splice variants of *Schistosoma mansoni* tropomyosin: Associations with immunity. Int. J. Parasitol..

[41] Ludolf F., Patrocínio P.R., Corrêa-Oliveira R., Gazzinelli A., Falcone F.H., Teixeira-Ferreira A., Perales J., Oliveira G.C., Silva-Pereira R.A. (2014). Serological screening of the *Schistosoma mansoni* adult worm proteome. PLoS Negl. Trop. Dis..

[42] Gnanasekar M., Salunkhe A.M., Mallia A.K., He Y.X., Kalyanasundaram R. (2009). Praziquantel affects the regulatory myosin light chain of *Schistosoma mansoni*. Antimicrob. Agents Chemother..

[43] Havercroft J.C., Huggins M.C., Dunne D.W., Taylor D.W. (1990). Characterization of Sm20, a 20-kilodalton calcium-binding protein of *Schistosoma mansoni*. Mol. Biochem. Parasitol..

[44] Mohamed M.M., Shalaby K.A., LoVerde P.T., Karim A.M. (1998). Characterization of Sm20.8, a member of a family of schistosome tegumental antigens. Mol. Biochem. Parasitol..

[45] Cohen C., Reinhardt B., Castellani L., Norton P.K., Stirewalt M. (1982). Schistosome surface spines are “crystals” of actin. J. Cell Biol..

[46] Sulbarán G., Alamo L., Pinto A., Marquez G., Méndez F., Padrón R., Craig R. (2015). An invertebrate smooth muscle with striated muscle myosin filaments. Proc. Natl. Acad. Sci. USA.

[47] Nelson M.R., Thulin E., Fagan P.A., Forsén S., Chazin W.J. (2002). The EF-hand domain: A globally cooperative structural unit. Protein Sci..

[48] Zhang Y., Taylor M.G., Johansen M.V., Bickle Q.D. (2001). Vaccination of mice with a cocktail DNA vaccine induces a Th1-type immune response and partial protection against *Schistosoma japonicum* infection. Vaccine.

[49] Del Mar Siles-Lucas M., Gottstein B. (2003). The 14-3-3 protein: A key molecule in parasites as in other organisms. Trends Parasitol..

[50] Foote M., Zhou Y. (2012). 14-3-3 proteins in neurological disorders. Int. J. Biochem. Mol. Biol..

[51] Carmona G., Göttig S., Orlandi A., Scheele J., Baüerle T., Jugold M., Kiessling F., Henschler R., Zeiher A.M., Dimmeler S. (2009). Role of the small GTPase Rap1 for integrin activity regulation in endothelial cells and angiogenesis. Blood.

[52] Schechtman D., Tarrab-Hazdai R., Arnon R. (2001). The 14-3-3 protein as a vaccine candidate against schistosomiasis. Parasite Immunol..

[53] Elzoheiry M., Da’dara A.A., Bhardwaj R., Wang Q., Azab M.S., El-Kholy E.-S.I., El-Beshbishi S.N., Skelly P.J. (2018). Intravascular *Schistosoma mansoni* cleave the host immune and hemostatic signaling molecule sphingosine-1-phosphate via tegumental alkaline phosphatase. Front. Immunol..

[54] Cesari I.M., Pujol F.H., Rodríguez M., Alarcón de Noya B. (1987). Possible use of *Schistosoma mansoni* enzymes as antigens for immunodiagnosis. Mem. Inst. Oswaldo Cruz..

[55] Rofatto H.K., Araujo-Montoya B.O., Miyasato P.A., Levano-Garcia J., Rodriguez D., Nakano E., Verjovski-Almeida S., Farias L.P., Leite L.C.C. (2013). Immunization with tegument nucleotidases associated with a subcurative praziquantel treatment reduces worm burden following *Schistosoma mansoni* challenge. PeerJ.

[56] Asseman C., Pancré V., Delanoye A., Capron A., Auriault C.A. (1994). Radioimmunoassay for the quantification of human ubiquitin in biological fluids: Application to parasitic and allergic diseases. J. Immunol. Methods.

[57] Majetschak M. (2011). Extracellular ubiquitin: Immune modulator and endogenous opponent of damage-associated molecular pattern molecules. J. Leukoc. Biol..

[58] Kanamura H.Y., Hancock K., Rodrigues V., Damian R.T. (2002). *Schistosoma mansoni* heat shock protein 70 p elicits an early humoral immune response in *S. mansoni* infected Baboons. Mem. Inst. Oswaldo Cruz..

[59] Zhou S., Jin X., Chen X., Zhu J., Xu Z., Wang X., Liu F., Hu W., Zhou L., Su C. (2015). Heat shock protein 60 in eggs specifically induces Tregs and reduces liver immunopathology in mice with *Schistosomiasis japonica*. PLoS ONE.

[60] Chen X., Li W., Li Y., Xu L., Zhou S., Zhu J., Xu Z., Liu F., Lin D., Hu F. (2017). Elevated serum antibody against *Schistosoma japonicum* HSP60 as a promising biomarker for liver pathology in schistosomiasis. Sci. Rep..

[61] Salvador-Recataà V., Greenberg R.M. (2012). Calcium channels of schistosomes: Unresolved questions and unexpected answers. WIREs Membr. Transp. Signal..

[62] Shalaby K.A., Yin L., Thakur A., Christen L., Niles E.G., LoVerde P.T. (2003). Protection against *Schistosoma mansoni* utilizing DNA vaccination with genes encoding Cu/Zn cytosolic superoxide dismutase, signal peptide-containing superoxide dismutase and glutathione peroxidase enzymes. Vaccine.

[63] Nordberg J., Arner E.S.J. (2001). Reactive oxygen species, antioxidants, and the mammalian thioredoxin system. Free Radic. Biol. Med..

[64] Kuntz A.N., Davioud-Charvet E., Sayed A.A., Califf L.L., Dessolin J., Arnér E.S.J., Williams D.L. (2007). Thioredoxin glutathione reductase from *Schistosoma mansoni*: An essential parasite enzyme and a key drug target. PLoS Med..

[65] Cao X., Hong Y., Zhang M., Han Y., Wu M., Wang X., Guo X., Li C., Lu K., Li H. (2014). Cloning, expression and characterization of protein disulfide isomerase of *Schistosoma japonicum*. Exp. Parasitol..

[66] Damonneville M., Auriault C., Pierce R.J., Capron A. (1982). Antigenic properties of *Schistosoma mansoni* aminopeptidases: Evolution during the development in mammalian hosts. Mol. Biochem. Parasitol..

[67] McCarthy E., Stack C., Donnelly S.M., Doyle S., Mann V.H., Brindley P.J., Stewart M., Day T.A., Maule A.G., Dalton J.P. (2004). Leucine aminopeptidase of the human blood flukes, *Schistosoma mansoni* and *Schistosoma japonicum*. Int. J. Parasitol..

[68] Fernández-Delgado M., Cortéz J., Sulbarán G., Matos C., Incani R.N., Ballén D.E., Cesari I.M. (2017). Differential distribution and biochemical characteristics of hydrolases among developmental stages of *Schistosoma mansoni* may offer new anti-parasite targets. Parasitol. Int..

[69] Ma L., Li D., Yuan C., Zhang X., Ta N., Zhao X., Li Y., Feng X. (2017). SjCRT, a recombinant *Schistosoma japonicum* calreticulin, induces maturation of dendritic cells and a Th1-polarized immune response in mice. Parasit. Vectors.

[70] Tanaka T., Hosoi F., Yamaguchi-Iwai Y., Nakamura H., Masutani H., Ueda S., Nishiyama A., Takeda S., Wada H., Spyrou G. (2002). Thioredoxin-2 (TRX-2) is an essential gene regulating mitochondria-dependent apoptosis. EMBO J..

[71] Abath F.G., Xavier E.M., Allen R., Gomes Y.M., Lucena-Silva N., Baliza M., Simpson A.J. (2000). Characterization of Sm13, a tegumental antigen of *Schistosoma mansoni*. Parasitol. Res..

[72] Hall T.M., Joseph G.T., Strand M. (1995). *Schistosoma mansoni*: Molecular cloning and sequencing of the 200-kDa chemotherapeutic target antigen. Exp. Parasitol..

[73] Braschi S., Castro-Borges W., Wilson R.A. (2006). Proteomic analysis of the schistosome tegument and its surface membranes. Mem. Inst. Oswaldo Cruz.

[74] Castro-Borges W., Dowle A., Curwen R.S., Thomas-Oates J., Wilson R.A. (2011). Enzymatic shaving of the tegument surface of live schistosomes for proteomic analysis: A rational approach to select vaccine candidates. PLoS Negl. Trop. Dis..

[75] Farias L.P., Tararam C.A., Miyasato P.A., Nishiyama Jr M.Y., Oliveira K.C., Kawano T., Verjovski-Almeida S., Leite L.C.C. (2011). Screening the *Schistosoma mansoni* transcriptome for genes differentially expressed in the schistosomulum stage in search for vaccine candidates. Parasitol. Res..

[76] Chalmers I.W., Fitzsimmons C.M., Brown M., Pierrot C., Jones F.M., Wawrzyniak J.M., Fernandez-Fuente N., Tukahebwa E.M., Dunne D.W., Khalife J. (2015). Human IgG1 responses to surface localized *Schistosoma mansoni* Ly6 family members drop following praziquantel treatment. PLoS Negl. Trop. Dis..

[77] Shao S., Sun X., Chen Y., Zhan B., Zhu X. (2020). Complement evasion: An effective strategy that parasites utilize to survive in the host. Front. Microbiol..

[78] Tanigawa C., Fujii Y., Miura M., Nzou S.M., Mwangi A.W., Nagi S., Hamano S., Njenga S.M., Mbanefo E.C., Hirayama K. (2015). Species-specific serological detection for schistosomiasis by serine protease inhibitor (SERPIN) in multiplex assay. PLoS Negl. Trop. Dis..

[79] Leontovyč A., Ulrychová L., O’Donoghue A.J., Vondrášek J., Marešová L., Hubálek M., Fajtová P., Chanová M., Jiang Z., Craik C.S. (2018). SmSP2: A serine protease secreted by the blood fluke pathogen *Schistosoma mansoni* with anti-hemostatic properties. PLoS Negl. Trop. Dis..

[80] Simpson A.J.G., Rumjanek F.D., Payares G., Evans W.H. (1981). Glycosyl transferase activities are associated with the surface membrane in adult *Schistosoma mansoni*. Mol. Biochem. Parasitol..

[81] Cass C.L., Johnson J.R., Califf L.L., Xu T., Hernandez H.J., Stadecker M.J., Yates J.R., Williams D.L. (2007). Proteomic analysis of *Schistosoma mansoni* egg secretions. Mol. Biochem. Parasitol..

[82] Mebius M.M., van Genderen P.J.J., Urbanus R.T., Tielens A.G.M., de Groot P.G., van Hellemond J.J. (2013). Interference with the host haemostatic system by schistosomes. PLoS Pathog..

[83] Gao H., Yu C.X. (2014). Enolase and parasitic infection. Zhongguo Xue Xi Chong Bing Fang Zhi Za Zhi.

[84] Figueiredo B.C., Da’dara A.A., Oliveira S.C., Skelly P.J. (2015). Schistosomes enhance plasminogen activation: The role of tegumental enolase. PLoS Pathog..

[85] Gao H., Xiao D., Song L., Zhang W., Shen S., Yin X., Wang J., Ke X., Yu C., Zhang J. (2015). Assessment of the diagnostic efficacy of enolase as an indication of active infection of *Schistosoma japonicum*. Parasitol. Res..

[86] Onile O.S., Calder B., Soares N.C., Anumudu C.I., Blackburn J.M. (2017). Quantitative label-free proteomic analysis of human urine to identify novel candidate protein biomarkers for schistosomiasis. PLoS Negl. Trop. Dis..

[87] Campbell C.H., Binder S., King C.H., Knopp S., Rollinson D., Person B., Webster B., Allan F., Utzinger J., Ame S.M. (2020). SCORE operational research on moving toward interruption of schistosomiasis transmission. Am. J. Trop. Med. Hyg..

[88] Tebeje B.M., Harvie M., You H., Loukas A., McManus D.P. (2016). Schistosomiasis vaccines: Where do we stand?. Parasit. Vectors.

[89] Crosnier C., Brandt C., Rinaldi G., McCarthy C., Barker C., Clare S., Berriman M., Wright G.J. (2019). Systematic screening of 96 *Schistosoma mansoni* cell-surface and secreted antigens does not identify any strongly protective vaccine candidates in a mouse model of infection. Wellcome Open Res..

[90] Dean D.A., Murrell K.D., Xu S.T., Mangold B.L. (1983). Immunization of mice with ultraviolet-irradiated *Schistosoma mansoni* cercariae: A re-evaluation. Am. J. Trop. Med. Hyg..

[91] Fukushige M., Mitchell K.M., Bourke C.D., Woolhouse M.E., Mutapi F. (2015). A meta-analysis of experimental studies of attenuated *Schistosoma mansoni* vaccines in the mouse model. Front. Immunol..

[92] Bergquist N.R., Colley D.G. (1998). Schistosomiasis vaccines research to development. Parasitology.

[93] Wilson R.A. (2012). Proteomics at the schistosome-mammalian host interface: Any prospects for diagnostics or vaccines?. Parasitology.

[94] Curwen R.S., Ashton P.D., Johnston D.A., Wilson R.A. (2004). The *Schistosoma mansoni* soluble proteome: A comparison across four life-cycle stages. Mol. Biochem. Parasitol..

[95] Barratt M.D. (1995). Quantitative structure-activity relationships for skin permeability. Toxicol. Vitr..

[96] Rutherford B.J., Dahl R.H., Price R.E., Szmidt H.L., Benke P.I., Mukhopadhyay A., Keasling J.D. (2010). Functional genomic study of exogenous n-butanol stress in *Escherichia coli*. Appl. Environ. Microbiol..

[97] Fletcher E., Pililzota T., Davies P.R., McVey A., French C.E. (2016). Characterization of the effects of *n*-butanol on the cell envelope of *E. coli*. Appl. Micriobiol. Biotechnol..

[98] Cook R.M., Carvalho-Queiroz C., Wilding G., LoVerde P.T. (2004). Nucleic acid vaccination with *Schistosoma mansoni* antioxidant enzyme cytosolic superoxide dismutase and the structural protein filamin confers protection against the adult worm stage. Infect. Immun..

[99] Sayed A.A., Cook S.K., Williams D.L. (2006). Redox balance mechanisms in *Schistosoma mansoni* rely on peroxiredoxins and albumin and implicate peroxiredoxins as novel drug targets. J. Biol. Chem..

[100] Mourão M.D.M., Dinguirard N., Franco G.R., Yoshino T.P. (2009). Role of the endogenous antioxidant system in the protection of *Schistosoma mansoni* primary sporocysts against exogenous oxidative stress. PLoS Negl. Trop. Dis..

[101] Hinks J., Wang Y., Matysik A., Kraut R., Kjelleberg S., Mu Y., Bazan G., Wuertz S., Seviour T. (2015). Increased microbial butanol tolerance by exogenous membrane insertion molecules. ChemSusChem.

[102] Nicolaou S.A., Gaida S.M., Papoutsakis E.T. (2010). A comparative view of metabolite and substrate stress and tolerance in microbial bioprocessing: From biofuels and chemicals, to biocatalysis and bioremediation. Metab. Eng..

[103] Aragon A.D., Imani R.A., Blackburn V.R., Cupit P.M., Melman S.D., Goronga T., Webb T., Loker E.S., Cunningham C. (2009). Towards an understanding of the mechanism of action of praziquantel. Mol. Biochem. Parasitol..

[104] Dunne D.W., Webster M., Smith P., Langley J.G., Richardson B.A., Fulford J., Butterworth A.E., Sturrock R.F., Kariuki H.C., Ouma J.H. (1997). The isolation of a 22 kDa band after SDS-PAGE of *Schistosoma mansoni* adult worms and its use to demonstrate that IgE responses against the antigen(s) it contains are associated with human resistance to reinfection. Parasite Immunol..

[105] Labateya N., Thomson D.M., Durko M., Shenouda G., Robb L., Scanzano R. (1987). Extraction of human organ-specific cancer neoantigens from cancer cells and plasma membranes with 1-butanol. Cancer Res..

[106] Liao S.K., Smith J.W., Kwong P.C. (1984). Selective extraction by 1-butanol of surface glycoprotein antigens from human melanoma cells. Cancer Immunol. Immunother..

[107] Akaberi M., Emami S.A., Vatani M., Tayarani-Najaran Z. (2018). Evaluation of antioxidant and anti-melanogenic activity of different extracts of aerial parts of *N. sintenisii* in murine melanoma B16F10 Cells. Iran. J. Pharm. Res..

[108] Noya O., Fermín Z., Alarcón de Noya B., Losada S., Colmenares C., Hermoso T. (1995). Humoral immune response of children with chronic schistosomiasis. Isotype recognition of adult worm antigens. Parasite Immunol..

[109] Bradford A. (1976). A rapid and sensitive method for the quantitation of micrograms quantities of protein utilizing the principle of protein-dye binding. Annal. Biochem..

[110] Laemmli U.K. (1970). Cleavage of structural proteins during the assembly of the head of bacteriophage T4. Nature.

