# Supplementary material for: Schistosoma mansoni Adult Worm Protective and Diagnostic Proteins in n-Butanol Extracts Revealed by Proteomic Analysis
Source: Pathogens. 2021 Dec 24;11(1):22. doi: 10.3390/pathogens11010022 (PMC8777762; doi:10.3390/pathogens11010022)
Supplement: Supplementary file 1 [file pathogens-11-00022-s001.zip › pathogens-1477694 revised final supplementary/TABLE S1.pdf]

TABLE S1 - Sm-AWBE PROTEINS IDENTIFICATION AFTER 1D-SDS/PAGE, BANDS GEL DIGESTION, MS ANALYSIS

Gel Section	Accession	Coverage (%)	Peptides	Description
**PTMSM1**	tr|A0A0R5RJE0|A0A0R5RJE0_SCHMA	16	2	Ubiquitin (Fragment) OS=Schistosoma mansoni OX=6183 PE=2 SV=1
	tr|Q8T9N5|Q8T9N5_SCHMA	24	3	Thioredoxin OS=Schistosoma mansoni OX=6183 GN=Smp_008070 PE=1 SV=1
**PTMSM2**	tr|G4M131|G4M131_SCHMA	56	10	Fatty acid binding protein OS=Schistosoma mansoni OX=6183 GN=Smp_095360.1 PE=3 SV=1
	P29498|FABP_SCHMA	56	10	14 kDa fatty acid-binding protein OS=Schistosoma mansoni OX=6183 PE=1 SV=1
	tr|A0A3Q0KEJ3|A0A3Q0KEJ3_SCHMA	32	4	Thioredoxin (Mitochondrial), Trx M OS=Schistosoma mansoni OX=6183 PE=4 SV=1
	tr|Q58HK3|Q58HK3_SCHMA	31	4	Mitochondrial thioredoxin OS=Schistosoma mansoni OX=6183 PE=2 SV=1
	tr|Q7KPG1|Q7KPG1_SCHMA	23	3	13 kDa tegumental antigen Sm13 OS=Schistosoma mansoni OX=6183 GN=GA157 PE=4 SV=1
	tr|G4LUD9|G4LUD9_SCHMA	22	5	Myosin regulatory light chain, putative OS=Schistosoma mansoni OX=6183 GN=Smp_132670.1 PE=4 SV=1
	tr|G4VCN0|G4VCN0_SCHMA	23	2	Putative fatty acid binding protein OS=Schistosoma mansoni OX=6183 GN=Smp_046800.2 PE=4 SV=1
	tr|B8Y6H3|B8Y6H3_SCHMA	10	2	DIF_5 OS=Schistosoma mansoni OX=6183 GN=Smp_105220 PE=2 SV=1
	tr|Q26593|Q26593_SCHMA	7	2	Protein disulfide-isomerase OS=Schistosoma mansoni OX=6183 PE=3 SV=1
**PTMSM5**	P42638|TPM2_SCHMA	22	5	Tropomyosin-2 OS=Schistosoma mansoni OX=6183 PE=2 SV=1
	tr|A0A5K4FBW8|A0A5K4FBW8_SCHMA	10	2	Tropomyosin-1 OS=Schistosoma mansoni OX=6183 PE=4 SV=1
	tr|Q26593|Q26593_SCHMA	6	3	Protein disulfide-isomerase OS=Schistosoma mansoni OX=6183 PE=3 SV=1
	tr|G4LYC3|G4LYC3_SCHMA	6	3	Protein disulfide-isomerase OS=Schistosoma mansoni OX=6183 GN=Smp_056760 PE=3 SV=1
	tr|G4M1D8|G4M1D8_SCHMA	11	3	Glutathione-S-transferase omega, putative OS=Schistosoma mansoni OX=6183 GN=Smp_152710.2 PE=3 SV=1
	Q26503|TPM_SCHHA	10	2	Tropomyosin OS=Schistosoma haematobium OX=6185 PE=2 SV=1
	P15845|SM20_SCHMA	15	2	20 kDa calcium-binding protein OS=Schistosoma mansoni OX=6183 GN=SM20 PE=2 SV=2
	tr|A0A5K4FCK1|A0A5K4FCK1_SCHMA	7	2	Calcium-binding protein, putative OS=Schistosoma mansoni OX=6183 PE=4 SV=1
**PTMSM6**	P42637|TPM1_SCHMA	17	5	Tropomyosin-1 OS=Schistosoma mansoni OX=6183 PE=2 SV=1
	Q26519|TPM_SCHJA	17	5	Tropomyosin OS=Schistosoma japonicum OX=6182 PE=2 SV=1
	P42638|TPM2_SCHMA	22	6	Tropomyosin-2 OS=Schistosoma mansoni OX=6183 PE=2 SV=1
	tr|G4VN74|G4VN74_SCHMA	22	6	Putative tropomyosin OS=Schistosoma mansoni OX=6183 GN=Smp_031770.4 PE=2 SV=1
	tr|Q26593|Q26593_SCHMA	4	2	Protein disulfide-isomerase OS=Schistosoma mansoni OX=6183 PE=3 SV=1
**PTMSM7**	tr|G4VN74|G4VN74_SCHMA	52	15	Putative tropomyosin OS=Schistosoma mansoni OX=6183 GN=Smp_031770.4 PE=2 SV=1

	P42638|TPM2_SCHMA	52	15	Tropomyosin-2 OS=Schistosoma mansoni OX=6183 PE=2 SV=1
	P42637|TPM1_SCHMA	38	11	Tropomyosin-1 OS=Schistosoma mansoni OX=6183 PE=2 SV=1
**PTMSM8**	P42638|TPM2_SCHMA	50	14	Tropomyosin-2 OS=Schistosoma mansoni OX=6183 PE=2 SV=1
	tr|G4VN74|G4VN74_SCHMA	50	14	Putative tropomyosin OS=Schistosoma mansoni OX=6183 GN=Smp_031770.4 PE=2 SV=1
	P42637|TPM1_SCHMA	42	11	Tropomyosin-1 OS=Schistosoma mansoni OX=6183 PE=2 SV=1
**PTMSM10**	P42637|TPM1_SCHMA	15	4	Tropomyosin-1 OS=Schistosoma mansoni OX=6183 PE=2 SV=1
	tr|G4VD36|G4VD36_SCHMA	15	4	Putative tropomyosin OS=Schistosoma mansoni OX=6183 GN=Smp_044010.2 PE=3 SV=1
	Q26519|TPM_SCHJA	15	4	Tropomyosin OS=Schistosoma japonicum OX=6182 PE=2 SV=1
	tr|G4LYU6|G4LYU6_SCHMA	7	3	Serpin, putative OS=Schistosoma mansoni OX=6183 GN=Smp_090080 PE=3 SV=1
	tr|Q26593|Q26593_SCHMA	8	4	Protein disulfide-isomerase OS=Schistosoma mansoni OX=6183 PE=3 SV=1
	tr|A0A3Q0KDW8|A0A3Q0KDW8_SCHMA	12	5	Calreticulin OS=Schistosoma mansoni OX=6183 PE=3 SV=1
**PTMSM12**	P42637|TPM1_SCHMA	17	4	Tropomyosin-1 OS=Schistosoma mansoni OX=6183 PE=2 SV=1
	tr|G4VD36|G4VD36_SCHMA	17	4	Putative tropomyosin OS=Schistosoma mansoni OX=6183 GN=Smp_044010.2 PE=3 SV=1
	Q26519|TPM_SCHJA	17	4	Tropomyosin OS=Schistosoma japonicum OX=6182 PE=2 SV=1
	tr|A0A3Q0KDW8|A0A3Q0KDW8_SCHMA	8	3	Calreticulin OS=Schistosoma mansoni OX=6183 PE=3 SV=1
	tr|G4LYU6|G4LYU6_SCHMA	5	2	Serpin, putative OS=Schistosoma mansoni OX=6183 GN=Smp_090080 PE=3 SV=1
	tr|A0A0R5RJF1|A0A0R5RJF1_SCHMA	9	2	Cu/Zn superoxide dismutase (Fragment) OS=Schistosoma mansoni OX=6183 PE=2 SV=1
	Q01137|SODC_SCHMA	8	2	Superoxide dismutase [Cu-Zn] OS=Schistosoma mansoni OX=6183 GN=SOD PE=1 SV=1
	tr|Q26593|Q26593_SCHMA	4	2	Protein disulfide-isomerase OS=Schistosoma mansoni OX=6183 PE=3 SV=1
	tr|G4VJ94|G4VJ94_SCHMA	5	2	Putative alkaline phosphatase OS=Schistosoma mansoni OX=6183 GN=Smp_155890 PE=3 SV=1
	tr|A8TKU6|A8TKU6_SCHMA	4	2	Alkaline phosphatase OS=Schistosoma mansoni OX=6183 PE=2 SV=1
**PTMSM 13-14**	tr|A0A5K4FCU9|A0A5K4FCU9_SCHMA	17	3	Tropomyosin-1 OS=Schistosoma mansoni OX=6183 PE=4 SV=1
	P42638|TPM2_SCHMA	14	4	Tropomyosin-2 OS=Schistosoma mansoni OX=6183 PE=2 SV=1
	tr|G4VN74|G4VN74_SCHMA	14	4	Putative tropomyosin OS=Schistosoma mansoni OX=6183 GN=Smp_031770.4 PE=2 SV=1
**PTMSM17**	tr|A8TKU6|A8TKU6_SCHMA	28	10	Alkaline phosphatase OS=Schistosoma mansoni OX=6183 PE=2 SV=1
	tr|B2D1S3|B2D1S3_SCHMA	13	5	Nucleotide pyrophosphatase/phosphodiesterase 5 OS=Schistosoma mansoni OX=6183 GN=NPP-5a PE=2 SV=1
	tr|C4QCZ2|C4QCZ2_SCHMA	12	5	Ecto-phosphodiesterase OS=Schistosoma mansoni OX=6183 GN=PDE PE=2 SV=1
	tr|A0A3Q0KDW8|A0A3Q0KDW8_SCHMA	10	4	Calreticulin OS=Schistosoma mansoni OX=6183 PE=3 SV=1
	tr|G4VN72|G4VN72_SCHMA	19	4	Putative tropomyosin OS=Schistosoma mansoni OX=6183 GN=Smp_031770.14 PE=4 SV=1

	P42638|TPM2_SCHMA	19	4	Tropomyosin-2 OS=Schistosoma mansoni OX=6183 PE=2 SV=1
	tr|P91803|P91803_SCHMA	8	3	Putative cytosol aminopeptidase (Fragment) OS=Schistosoma mansoni OX=6183 PE=2 SV=1
	tr|A0A3Q0KDV4|A0A3Q0KDV4_SCHMA	8	3	Leucine aminopeptidase (M17 family) OS=Schistosoma mansoni OX=6183 PE=4 SV=1
	tr|A0A5K4FCU9|A0A5K4FCU9_SCHMA	23	4	Tropomyosin-1 OS=Schistosoma mansoni OX=6183 PE=4 SV=1
	tr|G4VD36|G4VD36_SCHMA	15	4	Putative tropomyosin OS=Schistosoma mansoni OX=6183 GN=Smp_044010.2 PE=3 SV=1
	tr|G4LX87|G4LX87_SCHMA	5	2	Clumping factor A (Fibrinogen-binding protein A) (Fibrinogen receptor A), putative OS=Schistosoma mansoni OX=6183 GN=Smp_194050 PE=4 SV=1
	tr|G4VP56|G4VP56_SCHMA	1	2	200-kDa GPI-anchored surface glycoprotein OS=Schistosoma mansoni OX=6183 GN=Smp_017730 PE=4 SV=1
	tr|Q26607|Q26607_SCHMA	1	2	Surface protein (Fragment) OS=Schistosoma mansoni OX=6183 PE=2 SV=1
	tr|G4VCN8|G4VCN8_SCHMA	4	2	Dihydrolipoyl dehydrogenase OS=Schistosoma mansoni OX=6183 GN=Smp_046740 PE=3 SV=1
	tr|G4VQ58|G4VQ58_SCHMA	4	2	Phosphopyruvate hydratase OS=Schistosoma mansoni OX=6183 GN=Smp_024110 PE=3 SV=1
	Q27877|ENO_SCHMA	4	2	Enolase OS=Schistosoma mansoni OX=6183 GN=ENO PE=2 SV=1
	tr|A0A3Q0KC44|A0A3Q0KC44_SCHMA	7	2	Glycogenin-related OS=Schistosoma mansoni OX=6183 PE=4 SV=1
