# Supplementary material for: Schistosoma mansoni Adult Worm Protective and Diagnostic Proteins in n-Butanol Extracts Revealed by Proteomic Analysis
Source: Pathogens. 2021 Dec 24;11(1):22. doi: 10.3390/pathogens11010022 (PMC8777762; doi:10.3390/pathogens11010022)
Supplement: Supplementary file 1 [file pathogens-11-00022-s001.zip › pathogens-1477694 revised final supplementary/TABLE S2.pdf]

TABLE S2 - PROTEINS IDENTIFICATION AFTER IN-SOLUTION DIGESTION OF Sm-AWBE

Accession	Peptides	Avg. Mass	Description
P42638|TPM2_SCHMA	27	32696	Tropomyosin-2 OS=Schistosoma mansoni PE=2 SV=1
P42637|TPM1_SCHMA	25	32954	Tropomyosin-1 OS=Schistosoma mansoni PE=2 SV=1
tr|A8TKU6|A8TKU6_SCHMA	11	59374	Alkaline phosphatase OS=Schistosoma mansoni PE=2 SV=1
tr|D7R925|D7R925_SCHMA	11	59375	Alkaline phosphatase OS=Schistosoma mansoni GN=AP PE=2 SV=1
P29498|FABP_SCHMA	14	14848	14 kDa fatty acid-binding protein OS=Schistosoma mansoni PE=1 SV=1
Q06814|CALR_SCHMA	9	45398	Calreticulin OS=Schistosoma mansoni PE=2 SV=2
tr|Q26593|Q26593_SCHMA	6	54155	Protein disulfide-isomerase OS=Schistosoma mansoni PE=3 SV=1
P15845|SM20_SCHMA	6	17894	20 kDa calcium-binding protein OS=Schistosoma mansoni GN=SM20 PE=2 SV=2
tr|Q95VB7|Q95VB7_SCHMA	7	68226	Albumin OS=Schistosoma mansoni PE=2 SV=1
tr|C4QCZ2|C4QCZ2_SCHMA	12	52563	Ecto-phosphodiesterase OS=Schistosoma mansoni GN=PDE PE=2 SV=1
tr|P91803|P91803_SCHMA	7	56420	Putative cytosol aminopeptidase (Fragment) OS=Schistosoma mansoni PE=2 SV=1
P08418|HSP70_SCHMA	7	69875	Heat shock 70 kDa protein homolog OS=Schistosoma mansoni PE=2 SV=2
tr|Q7KPG1|Q7KPG1_SCHMA	5	11923	13 kDa tegumental antigen Sm13 OS=Schistosoma mansoni GN=GA157 PE=4 SV=1
Q26565|PPIA_SCHMA	6	17671	Peptidyl-prolyl cis-trans isomerase OS=Schistosoma mansoni PE=1 SV=1
Q27877|ENO_SCHMA	3	46995	Enolase OS=Schistosoma mansoni GN=ENO PE=2 SV=1
tr|Q8T9N5|Q8T9N5_SCHMA	3	11924	Thioredoxin OS=Schistosoma mansoni GN=Smp_008070 PE=1 SV=1
tr|B8Y6H3|B8Y6H3_SCHMA	4	14191	DIF_5 OS=Schistosoma mansoni GN=Smp_105220 PE=2 SV=1
Q01137|SODC_SCHMA	2	15721	Superoxide dismutase [Cu-Zn] OS=Schistosoma mansoni GN=SOD PE=1 SV=1
P53471|ACT2_SCHMA	4	41741	Actin-2 OS=Schistosoma mansoni PE=2 SV=1
tr|G4V5G7|G4V5G7_SCHMA	4	36511	Glycogenin-related OS=Schistosoma mansoni GN=Smp_008490 PE=4 SV=1
P09792|GST28_SCHMA	2	23820	Glutathione S-transferase class-mu 28 kDa isozyme OS=Schistosoma mansoni GN=GST28 PE=1 SV=1
tr|G4LUC3|G4LUC3_SCHMA	2	14129	Putative uncharacterized protein OS=Schistosoma mansoni GN=Smp_019350 PE=4 SV=1
Q26537|14332_SCHMA	2	24608	14-3-3 protein homolog 2 (Fragment) OS=Schistosoma mansoni PE=2 SV=1
tr|Q9U491|Q9U491_SCHMA	2	28754	14-3-3 epsilon OS=Schistosoma mansoni GN=Smp_034840.3 PE=2 SV=1
tr|Q95W36|Q95W36_SCHMA	2	28432	14-3-3 epsilon 2 OS=Schistosoma mansoni GN=Smp_002410 PE=2 SV=1
tr|G4V8A9|G4V8A9_SCHMA	2	28790	14-3-3 epsilon OS=Schistosoma mansoni GN=Smp_034840.2 PE=3 SV=1

Accession	Peptides	Avg. Mass	Description
Q26540|14331_SCHMA	2	28372	14-3-3 protein homolog 1 OS=Schistosoma mansoni PE=2 SV=1
tr|Q8MXA4|Q8MXA4_SCHMA	2	58435	Heat shock protein HSP60 (Fragment) OS=Schistosoma mansoni PE=2 SV=1
tr|Q963L7|Q963L7_SCHMA	2	203517	High voltage-activated calcium channel Cav1 OS=Schistosoma mansoni PE=2 SV=1
tr|G4VFV4|G4VFV4_SCHMA	2	20700	Putative rap1 and OS=Schistosoma mansoni GN=Smp_071250 PE=4 SV=1
