# Supplementary material for: Schistosoma mansoni Adult Worm Protective and Diagnostic Proteins in n-Butanol Extracts Revealed by Proteomic Analysis
Source: Pathogens. 2021 Dec 24;11(1):22. doi: 10.3390/pathogens11010022 (PMC8777762; doi:10.3390/pathogens11010022)
Supplement: Supplementary file 1 [file pathogens-11-00022-s001.zip › pathogens-1477694 revised final supplementary/TABLE S3.pdf]

**Table S3**. Known molecular and functional properties of the proteomics-identified Sm-AWBE proteins*

| Gene Name | Protein name | Description/Properties/Functions | Observations |
|---|---|---|---|
| Smp_002410 | 14-3-3 epsilon 2 | The 14-3-3 proteins compose a family of conserved regulatory molecules that binds a multitude of functionally diverse signaling proteins, including kinases, phosphatases, transmembrane receptors, etc. [49]. 14-3-3 proteins have been assessed in vaccination trials against helminth infections with different findings [52, 48]. | Antibodies against 14-3-3- proteins are induced in the course of natural *S. mansoni* infections. Experimental vaccinations led to partial 25-46% protection in terms of adult worm burden reduction [52] |
| Smp_005350 | 20 kDa calcium-binding protein [SM20] | $Ca^{2+}$-binding protein expressed in schistosomula and adult worm; it is associated with the tegumental membrane, not present in eggs; it has homology to calmodulin, troponin, myosin. | SM20 is a *S. mansoni* tegumental surface antigen [43, 44]. |
| Smp_008070 | Thioredoxin [Trx] | $Ca^{2+}$ binding Trx has protein disulfide oxidoreductase activity; it contains one Trx domain. It is produced as a response to ROS and protects schistosomes from oxidative stress. It plays a role in redox signaling and effects cell redox homeostasis. Acts as antioxidant by facilitating the reduction of other proteins by cysteine thiol-disulfide exchange [63]. | Antioxidant *S. mansoni* enzyme, redox homeostasis. Key drug target [64]. |
| Smp_008490 | Glycogenin-related | Transmembrane glycosyl transferase involved in Gluconeogenesis. Possible drug target. | Glycosyl transferases are on the *S. mansoni* surface [80]. Glycogenin was found by proteomics in *S. mansoni* egg excretions [81]. |
| Smp_008545 | Heat shock protein 60 [HSP60] | ATP/Nucleotide binding chaperone with protein folding activity; stress response. HSP60 induces regulatory T cells and reduces liver pathology in mice infected with *S. japonicum* [59]. | HSP60 is immunomodulatory [59]; it is antigenic: there is elevated serum antibody against HSP60 in *S. japonicum*-infected humans [60]. |
| Smp_009580 | Ubiquitin [UB] | Increased extracellular concentrations of UB are found in patients with schistosomiasis [56] UB is a post-translational protein modifier, involved in protein ubiquitination: it has other important intracellular and extracellular functions [57]. | Extracellular UB immunonodulate the immune response and prevent exuberant inflammation [57]. It is immunorecognized by infected people, especially through an IgE response [28]. |
| Smp_009760 | 14-3-3 protein homolog 1 | The 14-3-3 proteins compose a family of conserved regulatory molecules that binds a multitude of functionally diverse signaling proteins, including kinases, phosphatases, transmembrane receptors, etc. [49, 50]. 14-3-3 proteins have been assessed in vaccination trials against helminth infections with different findings [52, 48]. | Antibodies against 14-3-3- proteins are induced in the course of natural *S. mansoni* infections. Experimental vaccinations led to partial 25-46% protection in terms of adult worm burden reduction [52]. |
| Smp_024110 | Enolase/Phosphopyruvate hydratase [ENO] | $Mg^{2+}$-phosphopyruvate hydratase is a cytosolic homodimeric enzyme (enolase family) involved in the glycolytic process. Present also on the *S. mansoni* tegument surface (from where it may cause plasminogen activation) [84]. | SmENO is recognized as antigen by “Infected” and “Normal Endemic” individuals [41]. SjENO is the most abundant ES antigen of *S. japonicum* and can be used to show active infection and to evaluate treatments [83, 85]. Biomarker for detecting schistosomiasis in urine |

| | | | |
|---|---|---|---|
| | | | [86]. |
| Smp_030000 | Leucine aminopeptidase (M17)/putative cytosol aminopeptidase [LAP] | LAP (M17 family); $Mn^{2+}$ aminopeptidase (metalloexopeptidase) activity. Removal of N-terminal amino acids from peptides. Present in the tegumental surface, cytoplasm, and gastrodermis of adult *S. mansoni* worms [67]. | LAP was shown to be an antigenic enzyme [66]. Found also in developmental stages of *S. mansoni* [68]. |
| Smp_030370 | Calreticulin [CALR] | $Ca^{2+}$-binding chaperone-lectin protein promoting folding, oligomeric assembly and quality control in the ER. CALR interacts with monoglucosylated proteins synthesized in the ER (Uniprot). | *S. mansoni* CALR is responsible for the high levels of protective immunity induced by radiation-attenuated schistosoma cercariae or schistosomula [69]. |
| Smp_031770 | Tropomyosin-2 [TPM-2] | TPM-2 is a homodimeric protein which in association with troponin plays a central role in the $Ca^{2+}$- dependent regulation of muscle contraction. Stabilizes actin filaments and control their interactions with actin-binding proteins. Associated with the tegument surrounding the sporocyst (Uniprot). | *S. mansoni* TPM-2 is highly antigenic and it induces an IgE response [40]. |
| Smp_034840 | 14-3-3 protein homolog 2 [14-3-3 protein epsilon] | 14-3-3 phosphoserine/phosphothreonine-binding proteins bind a multitude of partners (over 200 proteins). 14-3-3 proteins participate in phosphorylation-dependent protein–protein interactions. Through binding to their target proteins, 14-3-3 proteins participate in the regulation of a wide range of biological processes [50]. The 14-3-3 protein family has been previously assessed in vaccination trials against helminth infections with different findings [52, 49, 48]. | Antibodies against 14-3-3- proteins are induced in the course of natural *S. mansoni* infections [52]. Experimental vaccinations led to partial 25-46% protection in terms of adult worm burden reduction [52, 48]. |
| Smp_037530 | Mitochondrial thioredoxin [Mt Trx] | Mitochondrial thioredoxin or Thioredoxin-2 (TRX-2) is an essential gene regulating mitochondria-dependent apoptosis [70]. | Other functions unknown/ Uncharacterized *S. mansoni* proten. |
| Smp_040130 | Peptidyl-prolyl *cis-trans* isomerase [PPIA] | Adult worm *S. mansoni* PPIA (cyclophilin A, SmCyP A, 17-19 kDa) catalyzes the *cis-trans* isomerization of proline imidic peptide bonds in oligopeptides; they accelerates the folding of proteins. Found in the tegument, gut epithelium, muscle layers, in the interior of the parasite. Also in the secretome [29]. | Drug target: inhibited by cyclosporine A (CsA) [30]. PPIA immunomodulates the host immune system by altering dendritic and T cells function *in vitro* and by inducing a T cell regulatory phenotype, regulating antigen presenting cells [29]. |
| Smp_044010 | Tropomyosin-1 [TPM-1] | TPM-1 (homodimer) in association with troponin plays a central role in the $Ca^{2+}$- dependent regulation of muscle contraction. Stabilizes actin filaments and control their interactions with actin-binding proteins. Expressed at much higher levels in the adult worm stage as compared to the cercariae and the egg stages. | Involved in muscle contraction (Uniprot). TPM-1 is highly antigenic and induces IgE response [40]. |
| Smp_046600 | Actin [s] (Actin-1, Actin-2) | ATP/Nucleotide binding proteins. Actin(s) are highly conserved proteins hat play central role in many cell functions (cytoskeleton, cell motility, cell shape and polarity, regulation of transcription). The tegumental surface in adult worms has spines formed by crystallized actin molecules [45]. | *S. mansoni* actin is recognized by sera from "Normal Endemic" individuals and also by Sm-AWBE-vaccinated mice [17]. |
| Smp_046740 | Dihydrolipoyl dehydrogenase [DLD] | DLD active site is a redox-active disulfide bond. FAD cofactor binding (Uniprot). | Essential mitochondrial enzyme playing a vital role in energy metabolism. |

| | | | |
|---|---|---|---|
| Smp_054160 | Glutathione S-transferase class-mu 28 kDa isozyme [GST28] | GST28 (homodimer) conjugates GSH to a wide number of exogenous and endogenous hydrophobic electrophiles (GSH tranferase activity). GST isoenzymes play central role in the parasite detoxification system [35]. | Sm-GST28 is antigenic in humans and protective in mice, rats, hamsters, and baboons [31, 32, 33, 35, 36]. Synthetic Sm-GST28 constructs were protective against *S. mansoni* infection in rats and mice, and induced an IgE response [32]. |
| Smp_056760 | Protein disulfide-isomerase [PDI] | PDI activity is present in ER (SmERp60); it catalyzes the rearrangement of -S-S- bonds in proteins. Involved in cell redox homeostasis. | Secreted enzyme involved in rearrangement of -S-S- bonds in proteins and redox homeostasis; potential vaccine candidate against *S. japonicum* [65]. |
| Smp_071250 | Putative rap1 and [Rap1] | GTP binding. Membrane bound small GTPase mediating signal transduction. Small Rap1 GTPase found implicated in the control of cell adhesion in a variety of cell types [Uniprot, 51]. Possible drug target. | Involved in signal transduction related to cell adhesion [51]. Other functions Unknown/ Uncharacterized *S. mansoni* protein. |
| Smp_090080 | Serpin, putative | Highly reactive with sera of schistosomiasis patients, antigen for serological immunoassays and sero-epidemiological surveillance [78]. Extracellular or secreted serine protease inhibitor (estrogen regulated protein, EP45 type) [79]. | *S. mansoni* secreted antigen, used for sero-epidemiological surveillance [78, 79]. |
| Smp_095360 | 14 kDa fatty acid-binding protein [FABP/Sm14] | 14 kDa fatty acid-binding protein (FABP/Sm14); involved in the cytoplasmic transport of fatty acids such as arachidonic, oleic, palmitic and linolenic acids (*in vitro*) [37]. Schistosomes have a high requirement for lipids, they are not able to synthesize fatty acids and sterols *de novo*. Thus, they must acquire host lipids. FABP is present in cytoplasm, tubercles, muscle layers and body. | FABP/Sm14 is an antigen that has been used as vaccine against *S. mansoni* [38]. |
| Smp_105220 | DIF_5 (CD59-like, SmLy6B] | Surface tegument *S. mansoni* CD59-like GPI-anchored protein (SmCD59.2, named also SmLy6B) [76]. CD59-proteins are inhibitors of complement membrane attacks [77]. | Mice immunized with the DIF-5 gene as a DNA vaccine showed 22% partial reduction in adult worm burden [75]. *S. mansoni* immunomodulatory protein (inhibitor of complement membrane attacks); might be involved in parasite's immune evasion [77]. |
| Smp_106930 | Heat shock 70 kDa protein homolog [HSP70] | ATP/Nucleotide binding, Belongs to the heat shock protein 70 family. Involved in response to stress. Possibly plays an important role in parasite development and pathogenesis (Uniprot), and in parasite adaptation to new host environments [21]. | HSP70 is a major *S. mansoni* surface antigen and immunogen in *S. mansoni* infections; it elicits early humoral immune response in *S. mansoni* infected baboons [58]. |
| Smp_132670 | Myosin regulatory light chain, putative (MRLC) | $Ca^{2+}$ binding protein that regulates myosin motility; activation of muscle contraction (Uniprot). | *S. mansoni* MRLC is a putative site for PZQ action [42]. Recognized by "Infected" and "Normal Endemic" individuals [41]. |
| Smp_135140 | High voltage-activated calcium channel Cav1 β (SmCavβvar) | Voltage-gated calcium channel activity; in plasma membrane. Disordered, polar and polyampholyte regions. | *S. mansoni* β(SmCavβvar) might be a drug target [61]. |
| Smp_152710 | Glutathione-S-transferase omega, | Multifunctional enzyme displaying significant glutathione-dependent dehydroascorbate | Drug target in other biological systems. |

| | | | |
|---|---|---|---|
| | putative [GST omega] | reductase and thiol transferase enzymatic activities [34]. | |
| Smp_153390 | Nucleotide pyrophosphatase/ phosphodiesterase 5 (NPP-5a)/Ecto-phosphodiesterase [PDE] | Integral transmembrane helix protein nucleotide pyrophosphatase/phosphodiesterase 5 (NPP-5) activity; phosphodiesterase (PDE) catalytic activity; NPP-5a has 100% identitiy with ecto-PDE (Uniprot). | *S. mansoni* surface tegumental membrane-bound type I PDE is immunogenic in natural infections [54, 7]. Immunization with tegument nucleotidases associated with a subcurative PZQ treatment reduces worm burden following *S. mansoni* challenge [55]. |
| Smp_155890 | Alkaline phosphatase [AP] | Integral transmembrane helix protein; also GPI-anchored. Relevant phosphomonoesterase activity in *S. mansoni* adult worms [14, 15,]. SmAP is a surface tegumental membrane glycoprotein marker. It has multiple molecular features and functions across *S. mansoni* life cycle stages [16]. SmAP has immunomodulatory activity [53]. | SmAP is highly antigenic; used in a specific immunoassay for schistosomiasis mansoni diagnosis [Alkaline Phosphatase Immunocapture Assay (APIA)] [3, 11, 5]. By hydrolyzing host plasma sphingosine-1-phosphate SmAP would control host inflammation and platelet aggregation [53]. |
| Smp_176200 | Superoxide dismutase [Cu-Zn] [SOD Cu-Zn] | Cu-Zn binding, antioxidant oxidoreductase, destroys radicals which are normally produced within the cells and which are toxic to biological systems. Present in cytoplasm. This enzyme binds 1 copper ion per subunit and 1 zinc ion per subunit. | Recognized as antigen by "Infected" and "Normal Endemic" individuals [41]. DNA vaccination with genes encoding Cu/Zn cytosolic superoxide dismutase, signal peptide-containing superoxide dismutase and glutathione peroxidase enzymes protected against *S. mansoni* [62]. |
| Smp_194050 | Clumping factor A (Fibrinogen-binding protein A) (Fibrinogen receptor A), putative [cflA] | Fibrinogen receptor A (Fibrinogen receptor A) binds fibrinogen (Uniprot). Hepatosplenic schistosomiasis patients are in a hypocoagulable and hyperfibrinolytic state, indicating that schistosomes interfere with the haemostatic system of their host; schistosomes produce antithrombotic or thrombolytic molecules [82]. | cflA would favours *S. mansoni* immune evasion by binding soluble fibrinogen [Uniprot] [82]. |
| Smp_195190 | 13 kDa tegumental antigen Sm13/GA157 | Sm13/GA157 is a *S. mansoni* surface 13 kDa tegumental antigen protein [71]. | Sm13/GA157 is an antigen recognized by antibodies from mice protectively vaccinated with adult *S. mansoni* worm tegumental membranes [71]. |

* Gene names and protein descriptions according to TABLES S1 and S2. Most protein information retrieved in UniProt [https://www.uniprot.org/uniprot/] and by manual literature mining.
